# Near-source wastewater surveillance of SARS-CoV-2, norovirus, influenza virus and RSV across five different sites in the UK

**DOI:** 10.1371/journal.pgph.0004397

**Published:** 2025-04-09

**Authors:** Jay C. Bullen, Mina Mohaghegh, Fatima Tahir, Charlotte Hammer, Jacob Sims, Frederico Myers, Lucas Eisinger, Ali Reza Kasmati, Claire F. Trant

**Affiliations:** 1 Untap Health, London, United Kingdom; 2 Cambridge Infectious Diseases, Department of Veterinary Medicine, University of Cambridge, Cambridge, United Kingdom; 3 Lifescience Dynamics, London, United Kingdom; The Chinese University of Hong Kong Faculty of Medicine, HONG KONG

## Abstract

By tracking infectious diseases through sewage, municipal-scale wastewater surveillance has provided early warnings of future COVID-19 hospitalisations, identified biases in diagnostic testing, and is rapidly expanding to a broader array of pathogens. Despite applications in the targeted delivery of local interventions, near-source wastewater surveillance has received less attention and we know little about the near-source time series dynamics of contrasting pathogens. To address this, we conducted wastewater surveillance at five sites for SARS-CoV-2 and two sites for norovirus GI, norovirus GII, influenza A virus, influenza B virus, and human respiratory syncytial virus (RSV A and RSV B). Sites were selected for contrasting functions: an office, charity centre, museum, university, and care home. The key findings are (1) near-source wastewater detections were linked to local events (staff sickness, enhanced cleaning, changing populations); (2) wastewater detections decreased in the order norovirus GII > norovirus GI > SARS-CoV-2 ≈ influenza A ≈ RSV A > influenza B ≈ RSV B; (3) correlation between near-source wastewater data and national surveillance data increases as a function of catchment size and viral prevalence (examples include the SARS-CoV-2 BA.4/BA.5 variant peak at a museum and wastewater tracking the winter norovirus season); (4) strong weekday periodicity in near-source wastewater SARS-CoV-2 detections, with the correlation against COVID-19 case numbers increasing when modelling variable lag times between faecal shedding onset and clinical diagnosis (R^2^ = 0.45 increases to 0.84-0.86); (5) a log-linear relationship between the frequency of wastewater SARS-CoV-2 detection and log(catchment size⋅viral prevalence) (R^2^ = 0.6914-0.9066). Finally, we propose two use cases. Firstly, for rare or high-risk pathogens, near-source wastewater sentinel systems provide early warning of outbreaks, achieving high frequency community coverage without behaviour change and at low cost versus diagnostic testing. Secondly, for endemic pathogens, near-source wastewater reveals long-term patterns and trends, the effectiveness of local policies, and community vulnerabilities.

## 1. Introduction

Wastewater has long been used as a sample medium for monitoring human health at the population scale [1]. With fast and sensitive nucleic acid amplification methods [2], wastewater-based epidemiology (WBE) was applied systematically in the 1990s during the World Health Organisation’s (WHO) Global Polio Eradication Initiative to help target vaccination programmes towards the most vulnerable communities [3]. WBE formally became part of the WHO’s recommended monitoring practices in 2003 [4] and contributed towards suppression of the 2013-2014 polio outbreaks in Israel [5]. The 2010’s saw significant development in WBE for chemical compounds: pharmaceuticals, narcotics (especially opioids), and other chemical biomarkers [6,7]. Use cases for chemical WBE include identifying consumption patterns at weekday, seasonal and longer timescales [8], estimating total consumption and market value [9], and geographical identification of vulnerable communities [7,10].

During the COVID-19 pandemic many countries established wastewater surveillance programmes, to support the traditional surveillance methods which had relied upon the diagnostic testing of individuals (e.g., using lateral flow devices (LFD) to detect antigens or reverse transcription polymerase chain reaction (RT-PCR) to detect ribonucleic acids (RNA)). Municipal-scale wastewater surveillance aimed to support traditional surveillance in multiple ways: cost-effective coverage of the whole population, early warning of outbreaks, and identification of clinical testing biases.

Firstly, municipal wastewater surveillance offers cost-effective coverage of large populations. Achieving representative coverage of a large population through diagnostic tests administered to individuals is costly both in reagents and labour. Wastewater is already a pooled sample, containing contributions from many individuals. Consequently, by administering a single test on a wastewater sample, population coverage is achieved using fewer resources. For instance, in England, 74% of the population was represented using just a few hundred samples, collected from wastewater treatment works [11]. Sensitive nucleic acid amplification techniques such as RT-qPCR can detect viruses even when prevalence is low. For instance, SARS-CoV-2 RNA has been detected in wastewater with as few as 2 people in 10,000 infected [12]. Depending upon wastewater catchment size and the frequency of testing, long-term surveillance programmes can cost <$1 USD per person per year (e.g., 100,000 people in a catchment, tested twice per week) [3].

Secondly, municipal-scale wastewater surveillance can provide an early warning of outbreaks. Multiple studies have reported increases in wastewater SARS-CoV-2 RNA anticipating increases in reported COVID-19 case numbers by 6 days [13] and hospitalisations by multiple weeks [14]. These lead times are determined by multiple factors. (i) Viral shedding into wastewater via faeces typically begins soon after infection, whilst it may take several days for symptoms to appear [3]. (ii) Symptomatic individuals may be undiagnosed for several days depending upon the availability of clinical testing [3,15]. (iii) The turnaround time between sample collection and sample results varies according to clinical or wastewater lab capacity, whether facilities are centralised or decentralised, and overall reporting pipelines [13]. When wastewater data leads clinical testing, it may function as an early warning system to support decisions such as enhancing lockdown measures or geographically targeting clinical testing capacity to where it is needed most [16].

Thirdly, municipal-scale wastewater surveillance can help compensate for testing biases. Wastewater samples are collected without any change in the behaviour of the general population (sampling is non-invasive). Consequently, wastewater data is free from many of the testing biases that appear in traditional diagnostic surveillance [17,18]. Examples include when there are changes to the availability of tests or the public appetite to seek testing. When COVID-19 LFD tests ceased to be available free-of-charge to the British public in March 2022, the proportion of people intending to continue using these tests fell from ~50% to 10% [19]. Wastewater surveillance has been used not just to indicate where diagnostic data under-reports true case numbers [20], but also to evaluate the success of public health interventions, such as lockdowns or shelter-in-place measures [3,21].

New applications for municipal wastewater surveillance continue to develop, including (a) the detection of new COVID-19 variants of concern, especially at national borders (airports and ferry ports) [3], (b) environmental surveillance of Mpox in catchments without reported cases [22], and (c) understanding the interconnections between public health, animal health, and the natural environment (One Health) [23], of which a prime example is the identification of the major pathways by which antimicrobial resistance (AMR) genes are released into the environment [24].

Use cases for wastewater surveillance are less established at the near-source scale. Near-source surveillance comprises the analysis of samples collected upstream of the sewer network, at building level [25]. This method has been piloted at apartment blocks [26], hospitals [27], universities [28] and schools [29]. During the COVID-19 pandemic, near-source wastewater detections were followed up with shelter-in-place requests issued to at-risk communities whilst building-level surge testing was conducted [28]. Continued surveillance after isolating identified COVID-19 cases has been used to confirm whether any infected individuals remain in the community [26].

The actions taken upon detection of an outbreak depend on both the type of pathogen detected and the type of facility or environment being monitored. Firstly, for respiratory pathogens such as SARS-CoV-2, influenza virus or human respiratory syncytial virus (RSV), airborne transmission is the primary concern. Mitigating actions include cough etiquette, wearing masks, and increased air ventilation. For gastro-intestinal pathogens such as norovirus, *Campylobacter* spp. and *Escherichia coli* (E. Coli), priority may instead be given to cleaning, food hygiene, and hand washing. Secondly, the recommended actions will also depend upon the setting. For instance, the United Kingdom has separate guidelines for acute care facilities, social care, and cruise ships with regards to PPE, cleaning protocols, and how to isolate infected and vulnerable individuals [30–32].

To date, few studies have assessed near-source wastewater monitoring at multiple sites with different functions side-by-side, or assessed the importance of catchment size on the resulting data. Furthermore, few studies have compared the detection of respiratory and gastro-intestinal viruses in near-source settings.

Our aim was to investigate the data and use cases provided by near-source wastewater surveillance for pathogens contrasting in both pathology and epidemiology (respiratory versus gastro-intestinal) at sites with a range of catchment sizes (50 to 2000 people per day) and a variety of functions (from care homes to museums). We first compared and contrasted SARS-CoV-2 wastewater patterns at five sites including a closed community (a care home), communities with the same population returning most days (an office and a university) and more transitory public-facing communities (a museum and a charity centre). We chose SARS-CoV-2 as the benchmark pathogen for all five sites, due to its prevalence at the start of the study. For the final two sites, we expanded our test panel to seven pathogen targets, to assess how wastewater detection patterns would vary according to pathogen characteristics. We included four additional respiratory pathogens: influenza A virus (IAV), influenza B virus (IBV), RSV A (RSVA), and RSV B (RSVB). We selected influenza virus given its significant seasonal contributions to morbidity and mortality worldwide [33] with an estimated 4.8 million lost working days per year in the UK [34]. We selected RSV since it is one of the most common causes of acute lower respiratory tract infection (LRTI) in both young children and older adults [35]. RSV is linked to nearly 500,000 GP appointments [36] and 8,482 associated deaths [37] per season in the UK, predominantly amongst young children and the elderly. We selected norovirus GI and GII as examples of gastro-intestinal pathogens, where shedding profiles are typically longer and different mitigation actions are required. Norovirus (NoV) is one of the most common causes of gastro-intestinal disease, with nearly 700 million cases per year globally [38]. Between the seven pathogens, incubation times vary from 2.4-2.6 days for SARS-CoV-2 Omicron BA.5 [39,40] and 1 to 3 days for influenza [41] to just 1.2 days for norovirus [42]. Estimated R0 values (basic reproduction numbers) are 1.28 for influenza [43], 3.0 for RSV [44], and increase from 2 to 7 for norovirus (population-wide estimates versus during an outbreak) [45]. The viral loads and shedding profiles of these pathogens also vary considerably. Similar to contemporary SARS-CoV-2 variants, the viral loads of influenza A peak 2 days after infection, with a mean viral shedding duration of 4.8 days and undetectable results after 8 days [35] (wastewater signals might last a couple of days) [46]. Norovirus shedding profiles last longer, from 7 to 12 days [47].

Our objectives were:

(i) To conduct near-source wastewater surveillance campaigns at multiple sites, with different catchment sizes and serving different functions, comparing differences in the detection of up to seven different pathogens.
(ii) To evaluate the extent to which near-source wastewater detection patterns correlate with local observations (for instance staff sickness or positive diagnostic tests).
(iii) To evaluate the extent to which near-source wastewater detection patterns correlate with diagnostic results from national surveillance programmes.
(iv) To investigate the actionability of wastewater surveillance results and identify improvements for near-source wastewater surveillance use cases.

## 2. Materials and methods

### 2.1. Field work

Wastewater monitoring was conducted across five sites in the UK (summarised in Table 1, with further details in Supporting Information S1 Text). The first three sites (2022) were monitored for the presence of SARS-CoV-2 only, whilst norovirus, influenza virus and RSV were also monitored at sites 4 and 5 (2023).

**Table 1:** Summary of field work sites and corresponding sample analysis methods. The catchment size was estimated using data provided by site managers, e.g., head counts or reception logs. Parentheses indicate minimum and maximum daily headcounts.

Site	Function	Estimated catchment size (people per day)	Start date	Finish date	Sample preparation method	Target pathogens
1	Offices	80-110	2022-02-09	2022-03-02	No pre-concentration and magnetic beads extraction	SARS-CoV-2
2	Charity centre	50-60	2022-03-08	2022-04-14		
3	Museum	2150	2022-06-27	2022-07-20		
4	University	980 (580-1150)	2023-10-09	2023-12-08	Centrifugal ultrafiltration and mini spin column extraction	SARS-CoV-2, norovirus GI, norovirus GII, influenza A virus, influenza B virus, RSV A, RSV B
5	Care home	190	2023-10-03	2023-12-08		

The first site was an office at an engineering company in Southampton (February 2022) with 80 to 110 employees on site daily. Typical working hours were 8am to 5pm and composite wastewater samples were collected between 7am and 6pm.

The second site was a charity centre supporting elderly citizens (March-April 2022) with 2 to 3 staff members and 50-60 daily visitors per day (approximately 200 unique visitors per week). Activities included computer training, art classes, seated exercise, yoga, sewing, and shared lunches. The centre was open between 10am and 4pm and composite samples were collected between 10am and 5pm.

The third site was a multi-use space in central London (June-July 2022), including a museum, offices, restaurants, and cafes. The site had 6450 visitors per day. Composite wastewater samples were collected from approximately one third of the restrooms on site, between 8am and 8pm.

The fourth site was a university co-working space in the East of England (October-December 2023), containing a canteen and meeting rooms. The total daily headcount was nearly 1000 but decreased to 500 when term ended during the last two weeks of sampling. Composite wastewater samples were collected between 7am and 7pm.

The fifth site was a residential care home in Berkshire (October-December 2023). The site had ~50 residents and 100 members of staff. The site also included a nursery, with ~30 children and 8 members of staff. Composite wastewater samples were collected, covering the entire site, between 7am and 11pm.

Composite samples were collected using a Hach AS950 autosampler (sites 1-4) and a Teledyne ISCO 6712 autosampler (site 5), producing one aggregate sample per day. Sampling intervals were optimised for battery life (6 minutes for Hach AS950 and 20 minutes for Teledyne ISCO 6712). Since the autosampler pumped for <30 seconds at each sampling event, <2.5-8% wastewater flow was likely sampled. The majority of elevated post-flush flow would be missed, however studies indicate that viral RNA persists in near-source sewer environments at significant concentrations for significant timescales during the post-flush period [48]. Samples were transported for laboratory analysis using a portable fridge. Samples were characterised for nitrogen content and pH as described in Supporting Information S1 Text. Samples were stored at 4 °C for nucleic acid extraction within 24 hours, and archived at -20 °C.

### 2.2. Nucleic acid concentration and extraction

During 2022, nucleic acids were extracted from samples collected at sites 1 and 2 using the LuminUltra SARS-CoV-2 wastewater kit and protocol (GCRNA-SARSWW-96C) [49]. Samples from site 3 were analysed using the LuminUltra SARS-CoV-2 wastewater plus kit and protocol (GCRNA-SARSWWPlus-48C, a replacement product with minor updates). This is a magnetic bead-based extraction method using 1 mL of wastewater sample, yielding 100 μL of eluted RNA in nuclease-free water.

During 2023, nucleic acids were extracted from samples collected at sites 4 and 5 using a centrifuge-based method, consisting of ultrafiltration followed by silica membrane extraction. Briefly, after heating 30 mL wastewater sample aliquots at 65 °C for 30 minutes, samples were centrifuged at 8194 rpm for 30 minutes. The pellet was discarded, and the supernatant then loaded in aliquots into a Millipore Amicon centrifugal ultrafilter (100 kDa, 15 mL capacity). The sample was ultrafiltered via centrifugation at 8194 rpm to yield a final volume of 300 μL retentate (from the initial 30 mL wastewater sample). After resuspension via pipetting, the retentate was added to 1 mL of pre-heated PM1 buffer at 55 °C. The Qiagen RNeasy PowerWater Kit method was then used according to the manufacturer’s protocol to yield 150 μL eluted RNA in nuclease-free water.

Eluted nucleic acids were analysed immediately or stored at -80 °C for up to two days prior to analysis. Further details on both extraction methods are provided in Supporting Information S1 Text.

### 2.3. Quantitative RT-PCR method

SARS-CoV-2 was detected using RT-qPCR assays from LuminUltra, targeting the N2 and E genes for sites 1 to 3 (using the GCRNA-SARSWW-96C kit) [50] and the N1 gene for sites 4 and 5 (using the upgraded GCRNA-SARSWWPlus-48C kit). SARS-CoV-2 RNA was detected in the FAM channel, and an internal PCR amplification control was included in the Cy5 channel. The RT-qPCR test was conducted using a LuminUltra GeneCount 16 portable qPCR instrument [49]. The positive control was a cDNA standard.

All other pathogen targets (norovirus*,* influenza virus and RSV) were detected using TaqMan microbe assays from Thermofisher (A39420) with amplification using a Strategene MX3000p qPCR instrument. Each assay was pooled, i.e., targeting multiple genes from the given pathogen within a single reaction, simultaneously detected in the FAM channel. Negative extraction controls were performed using deionised water and negative PCR controls were performed using RNase free water. Positive controls were performed using synthetic RNA standards from ATCC. Calibration curves to convert between C_t_ values and gene copy numbers were obtained through qPCR analysis of serial dilutions of the ATCC standards. Detection limits ranged between 1.9×10^3^ and 8.6×10^3^ gene copies L^-1^. An external calibration curve for SARS-CoV-2 was supplied by LuminUltra GeneCount 16 software and detection limits were 5.0×10^4^ and 2.6×10^3^ gene copies L^-1^ using (i) magnetic bead and (ii) centrifuge-based extraction methods respectively. Further details are provided in Supporting Information S1 Text.

### 2.4. National surveillance data

Publicly available national surveillance data from the UK government was used as an indicator of viral prevalence in the surrounding community. City-scale catchments were used for COVID-19 case numbers during 2022. Larger, regional catchments were used for the 2023 sites due to city-scale data showing significant noise (due to lower testing rates in 2023). The data available regarding the weekly number of norovirus laboratory reports [51] and weekly positivity rates for influenza virus and RSV [52] covered the entirety of England. Further details are available in Supporting Information S1 Text.

### 2.5. Data analysis

We first conducted a qualitative investigation of the near-source wastewater data set, to identify potential links firstly to local events (e.g., staff sickness) and secondly to trends in the wider community (national surveillance data).

To provide an initial quantitative understanding of the near-source wastewater data set, we then considered the independent variables of (a) virus prevalence, (b) weekday periodicity, and (c) catchment size. Firstly, we investigated whether SARS-CoV-2 wastewater detection was more likely on days with higher COVID-19 case numbers by calculating the mean, median, and interquartile range of COVID-19 case numbers using data from days (i) with, and (ii) without wastewater SARS-CoV-2 detection. Secondly, we explored weekday periodicity by calculating the total number of COVID-19 cases and wastewater SARS-CoV-2 detections for each week that a particular site was monitored. We then determined the proportion of each week’s total COVID-19 cases and wastewater SARS-CoV-2 detections that fell on a Monday, Tuesday, etc. We averaged the results across all weeks within a given site to provide a single weekday periodicity distribution for each site. We combined the results from each of the five sites into a single average weekday periodicity distribution. As described in the Supporting Information, we then considered a scenario where SARS-CoV-2 RNA is detected in wastewater before a clinical test is taken, and we used an exponential decay probability distribution to model a variable time lag between wastewater detection and clinical diagnosis. Thirdly, we considered catchment size by calculating log(cases per capita per day⋅catchment size), using the average daily case numbers and catchment size from each study period. We then correlated log(cases per capita per day⋅catchment size) against the total frequency of wastewater SARS-CoV-2 detections at each site.

We further quantified the relationship between wastewater detections and national surveillance data by calculating correlation coefficients and concordance rates. Wastewater surveillance has been used to make operational decisions at both same-day and longer timescales, e.g., the immediate issuing of shelter in place orders prior to surge testing [28] and the geographical targeting of vaccination programmes [3]. We therefore considered end-users consulting near-source wastewater data on both daily and weekly timescales. We first used (1) individual daily data points considered in isolation to the rest of the time series, and (2) five-point moving averages. We also considered (3) changes in the day-to-day measurement, both (i) using individual data points:


$$\Delta y_{t}=y_{t}-y_{t-1}$$ (1)

Where *t* is the day of interest and *y* is either the daily COVID-19 case rate, RNA concentration, or detection frequency (in this case, a binary 0% or 100%). And (ii) using the slope of the five most recent data points:


$$slope_{t}=\frac{5\sum_{i=t}^{t-4}\left(x_{i}y_{i}\right)-\sum_{i=t}^{t-4}x_{i}\sum_{i=t}^{t-4}y_{i}}{5\sum_{i=t}^{t-4}x_{i}^{2}-{\left(\sum_{i=t}^{t-4}x_{i}\right)}^{2}}$$ (2)

Where *x* is one of the five days sampled and is equivalent to *i*. Norovirus national surveillance data reported weekly aggregates only and consequently correlation and concordance were evaluated using (4) weekly averages, looking at week-to-week changes (using Equation 1).

Concordance rates were calculated exclusive of pairs with consecutive wastewater non-detects, using:


$$concordancerate\left(\%\right)=100\cdot \frac{numberofconcordantpairs}{numberofconcordantpairs+numberofdiscordantpairs}$$ (3)

And including pairs with consecutive wastewater non-detects:


$$concordancerate\left(\%\right)=100\cdot \frac{numberofconcordantpairs}{totalnumberofpairs}$$ (4)

Where concordant pairs are consecutive time series measurements with wastewater and clinical case numbers both moving in the same direction (increasing or decreasing), while discordant pairs have wastewater and clinical case numbers moving in opposite directions.

### 2.6. Ethics statement

The analysed wastewater samples were composites of human waste, greywater (e.g., bathrooms, kitchens, appliances) and rainwater, with between 50 and 2150 people in each sewer catchment (and at least 100 unique people per week). No identifying information could be gathered from these samples, ensuring anonymity [53]. Since the research did not involve individual human subjects, no ethics committee approval for this study was sought and written or verbal consent from individuals was not applicable. However, the study followed ethical research guidelines relevant to environmental health monitoring and permits and approvals for field sampling were acquired from the relevant authority at each site.

## 3. Results

### 3.1. Near-source wastewater surveillance of SARS-CoV-2 at five sites

#### 3.1.1. Wastewater detections linked to local events and national-scale trends.

**Fig 1:**
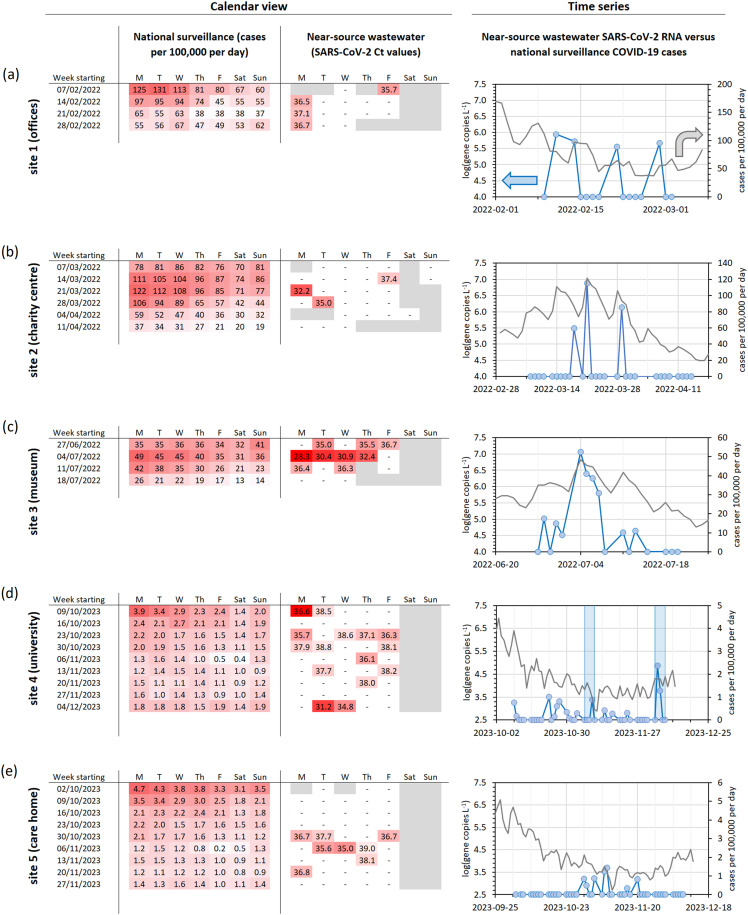
Timeline of 2022-2023 wastewater SARS-CoV-2 data alongside national surveillance reports. Heat maps present COVID-19 case numbers for the surrounding city or region (left) and wastewater C_t_ values (centre), whilst scatter plots (right) indicate wastewater SARS-CoV-2 gene copy numbers (blue) alongside daily COVID-19 case numbers for the surrounding area (grey). The blue shaded regions in the time series for site 4 (university) indicates weeks with staff sickness due to positive COVID-19 diagnosis or related symptoms.

Daily near-source wastewater SARS-CoV-2 RNA measurements from this study are presented alongside COVID-19 case numbers from the surrounding area, as reported by the UK government’s national surveillance programme (Fig 1). Calendar views are provided to highlight recurring weekday-dependent trends, and time series are provided to highlight long-term trends. In total, 153 wastewater samples were tested for SARS-CoV-2, with 22% of samples giving positive detections. This near-source SARS-CoV-2 detection rate is comparable to previous studies: 22% [54], 20-50% [26], 32% [29], 47% [48], <50% [55].

Of the sites tested in 2022 (sites 1-3), the strongest wastewater detections at each site were all found during weeks with the most reported COVID-19 cases. At site 1 (the office, Fig 1A), samples were analysed during a period of declining COVID-19 case numbers, after the Omicron BA.1 peak [56]. At this site, employees undertook SARS-CoV-2 tests each day using lateral flow devices. The office manager indicated that nearly all wastewater SARS-CoV-2 detections correlated to positive LFD results taken during routine screening by employees, with one exception. The exception occurred on a day when a group of external visitors, who were not asked to undergo LFD testing upon arrival, included COVID-19 positive individuals. At site 2 (the charity centre, Fig 1B) samples were analysed during the Omicron BA.2 peak [56] and the three positive wastewater detections are clustered around the same time as the national surveillance peak. Site 3 (the museum, Fig 1C) was sampled during the BA.4 and BA.5 variant outbreak [57] and shows a wastewater SARS-CoV-2 RNA peak with the same timing as national surveillance COVID-19 case numbers.

Samples were collected at sites 4 and 5 during a temporary minimum in COVID-19 case numbers (October-December 2023). COVID-19 cases had recently peaked due to a combination of XBB.1.5, XBB.1.16 and EG.5.1 variants, but were in decline at the start of October [58]. Cases reached a minimum in mid-November, and then began to rise again due to the emerging JN.1 strain (and to a lesser extent BA.2.86) [58]. At site 4 (the university, Fig 1D) wastewater SARS-CoV-2 detections occurred intermittently across the study period. Most detections occurred during the first four weeks, whilst COVID-19 cases continued to decline. Despite COVID-19 cases being at a minimum during week commencing 2023-11-06, there was one wastewater SARS-CoV-2 detection whilst a staff member was absent from work due to flu-like symptoms and another colleague reported a sore throat (influenza A virus was also detected twice this week, discussed later). There were no further wastewater detections or reports of staff sickness during the next three weeks, whilst COVID-19 cases remained at a minimum. COVID-19 cases began to increase from this temporary minimum during week commencing 2023-12-04 and there were two strong wastewater detections on 2023-12-05 and 2023-12-06 whilst multiple staff were off sick with COVID-19.

For sites 1 to 4, trends in near-source wastewater SARS-CoV-2 detection could be linked, at least partially, to changes in COVID-19 case numbers within the wider community (national surveillance). This was not true for site 5 (the care home, Fig 1E) where all near-source wastewater SARS-CoV-2 detections were grouped within the 3-week window when COVID-19 cases in the Southeast of England were at their lowest.

The greatest qualitative match between measured near-source wastewater SARS-CoV-2 RNA concentrations and COVID-19 cases was at site 3, a museum with the largest catchment size (approximately 2000 visitors per day) and the most open community, monitored during an outbreak of COVID-19. In contrast, there was no qualitative match between near-source wastewater and national surveillance data sets for the care home. Here, the catchment size was ten times smaller (190 people), the community was relatively closed-off (permanent residents with restricted visitors), the strictest hygiene and sanitation measures were used, and surveillance was conducted in the interval between COVID-19 variant outbreaks.

#### 3.1.2. Viral prevalence, catchment size, and weekday periodicity.

**Fig 2:**
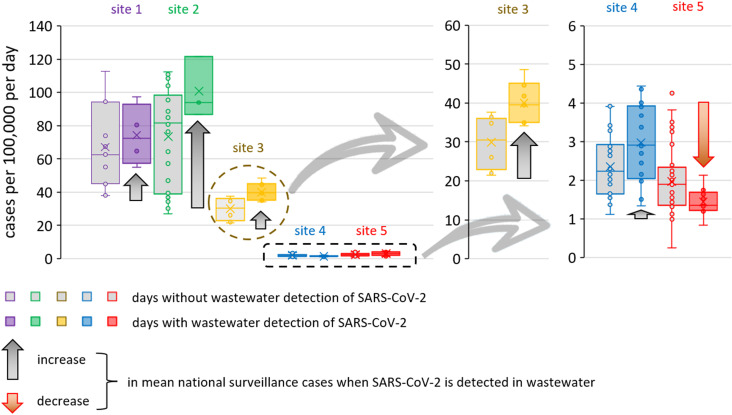
Comparison of COVID-19 case numbers on days with (coloured) and without (grey) wastewater detection of SARS-CoV-2 RNA. Minimum and maximum bounds indicate the first and third quartiles (25^th^ and 75^th^ percentiles) whilst box height indicates the interquartile range (IQR). Boxes are divided by the median (50^th^ percentile), whilst crosses indicate the mean. Whiskers indicate the minimum and maximum data points not considered to be outliers (1.5×IQR). The interquartile range is calculated exclusive of the median.

We next quantitatively investigated the links between near-source wastewater SARS-CoV-2 detections and case numbers in the wider community, considering the statistical distribution of national surveillance COVID-19 case numbers on days with and without wastewater SARS-CoV-2 RNA detection (Fig 2). At sites 1-4, COVID-19 case numbers tended to be greater on days with wastewater SARS-CoV-2 detection. The difference was smallest at site 1 where field work was conducted during a period of relatively low SARS-CoV-2 prevalence (after the Omicron BA.1 wave, with mean COVID-19 case numbers being just 11% greater on days with SARS-CoV-2 wastewater detection). The difference between mean COVID-19 case numbers on days with and without wastewater detection was greatest for sites 2 and 3 (+37% and +33% respectively). Both sites were sampled across a variant peak with a large relative change in COVID-19 case numbers, linked to a greater swing in the daily probability that we find individuals within the catchment population shedding SARS-CoV-2 RNA into wastewater. In contrast with all other sites, COVID-19 case numbers at site 5 (the care home) tended to be lower on days when SARS-CoV-2 was detected in the wastewater (-27% decrease). This agrees with earlier qualitative observations that this small and closed community shows the greatest divergence between local and national trends.

**Fig 3:**
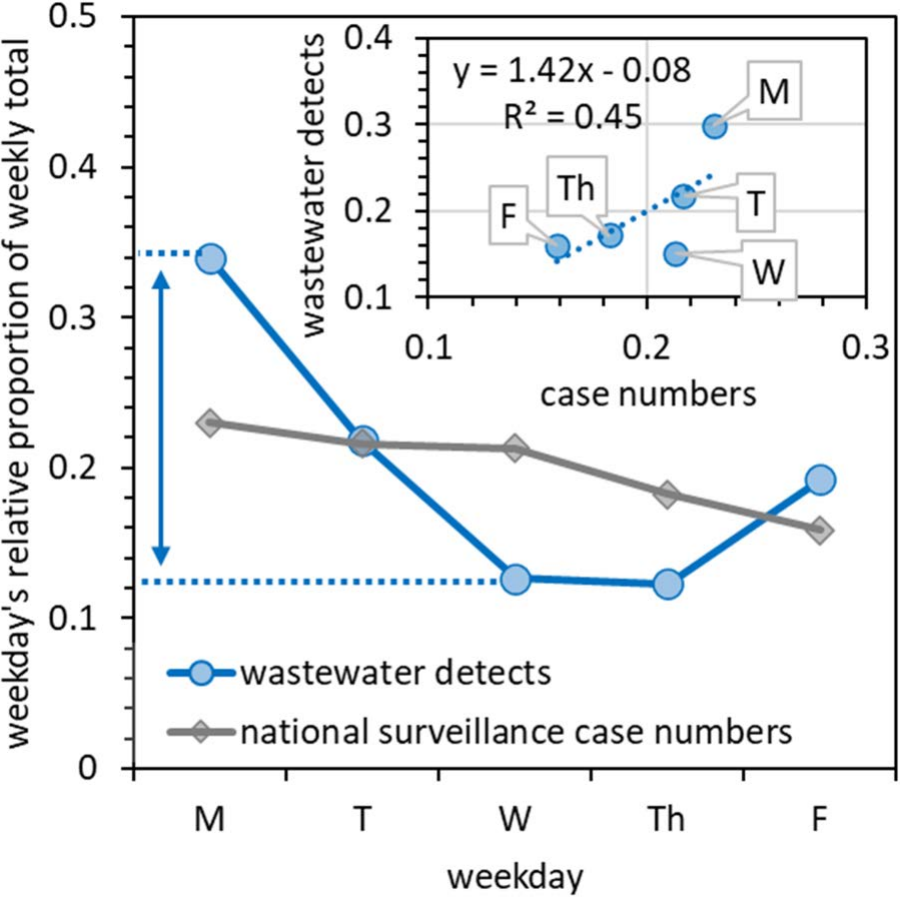
The proportion of SARS-CoV-2 wastewater detects and COVID-19 cases within each week that fall on a given weekday. Between 29 and 31 data points were available for each day of the week.

Fig 3 shows that reported COVID-19 case numbers tended to be greater at the start of each working week. Similar periodic patterns in the US have been assigned to testing biases (variability in weekday testing rates) as opposed to real differences in infection rates [59,60]. Reporting biases in the UK similarly include LFD tests being taken most frequently at the start of the working week (2020) [61]. However, we found that near-source wastewater detections were also more frequent at the start of the week (Fig 3) and often with greater RNA concentrations (Fig 1). In fact, the distribution of wastewater detection events showed greater weighting towards the start of the working week than the distribution of COVID-19 case numbers (Fig 3).

A Pearson correlation coefficient of R^2^ = 0.452 is observed between weekday-dependent COVID-19 case numbers and wastewater SARS-CoV-2 detection (Fig 3, inset figure). To explain why wastewater detection frequency exhibited more periodicity than COVID-19 case numbers, we considered the variability in the delay between when individuals are infected/present symptoms and when they undergo testing. With the simplification that wastewater peaks lead case numbers [62] by zero to seven days and that this lead time can be represented using an exponential decay probability distribution, the correlation coefficient between wastewater data and case numbers increases from R^2^ = 0.45 to 0.84-0.86 (Supporting Information, Fig B(d) in S1 Text). This improvement is achieved by optimisation of the exponential decay probability distribution, producing an average lag time between wastewater data and reported cases of 2.3 days (Fig B(b) in S1 Text). This is not unreasonable, given that elsewhere, wastewater lead times of 0-6 days have been reported [62].

These near-source wastewater surveillance results suggest that the high case numbers reported at the start of each week are not due to testing biases only. Instead, they also include a real component, with possible causes including social mixing over the weekend and differences between weekday and weekend population mobility [63]. The results also show that at least part of the correlation between near-source wastewater data and national surveillance data observed in Fig 2 is due to a weekday periodicity present in both data sets.

**Fig 4:**
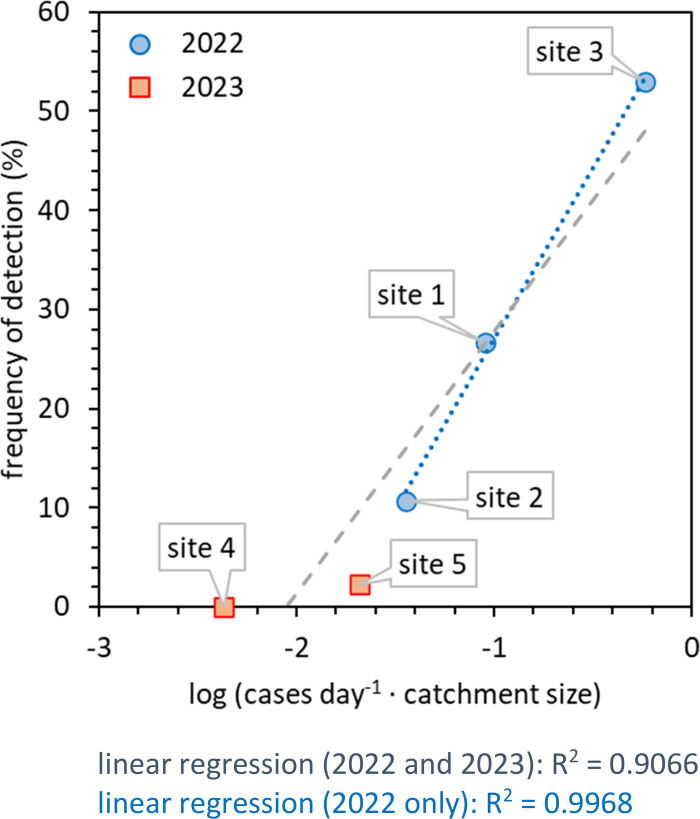
Correlation of wastewater SARS-CoV-2 RNA detection frequency with the sewershed catchment size. The product of COVID-19 case numbers per capita per day and catchment size varied by two orders of magnitude across the 5 sites, and consequently the logarithm was used. In contrast, the frequency of detection is displayed on a linear scale, with only a factor of four difference between the greatest and smallest detection frequencies.

Further analysis reveals that the frequency of wastewater SARS-CoV-2 detection is strongly linked to the catchment size at the sampling point (Fig 4). Firstly, a positive correlation between the wastewater SARS-CoV-2 detection frequency and the average COVID-19 case numbers at each of the five sites was not observed (Fig C(a) in S1 Text). In contrast, a positive correlation is observed between the frequency of wastewater detection and the catchment size, with a correlation coefficient of R^2^ = 0.5405 for all five sites, increasing to R^2^ = 0.9373 when only the three sites from 2022 are considered (Fig C(b) in S1 Text). This correlation further improves when catchment size is combined with case numbers, yielding R^2^ = 0.6914 when all five sites are considered and R^2^ = 0.9968 when only the three sites from 2022 are considered (Fig C(c) in S1 Text). Our SARS-CoV-2 detection limits improved between 2022 and 2023 due to changes to the sample processing procedure. Ignoring 2023 samples with SARS-CoV-2 RNA concentrations below the 2022 detection limit, the correlation improves to R^2^ = 0.9066 when all five sites are considered (Fig 4). These significant correlations highlight the importance of catchment size when comparing different sites, especially if risk thresholds are to be mapped onto wastewater results.

This analysis shows that despite the five sites all having very different functions, SARS-CoV-2 is reliably detected in near-source wastewater more frequently when catchments have more infected individuals available to shed into the wastewater. This is true both when considering catchments individually (Fig 2) and when comparing different catchments (Fig 4).

### 3.2. Norovirus, influenza virus and RSV surveillance at a university and care home

#### 3.2.1. Comparison of seven pathogens.

**Fig 5:**
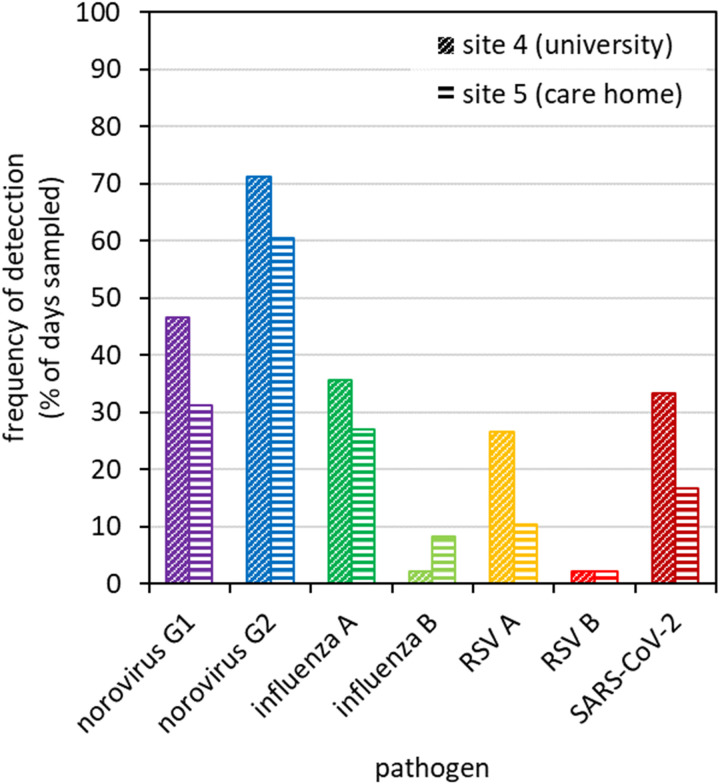
Frequency of wastewater detections for the different pathogens monitored during 2023 at site 4 (university, cross-hatched bars) and site 5 (care home, bars with horizontal stripes). Percentages indicate the proportion of all samples that gave a positive RT-qPCR result.

In 2023, the analytical panel was expanded to more viral targets, with contrasting pathology. The university (site 4) represented a larger catchment with an ‘open’ community, and the care home (site 5) represented a smaller catchment with a ‘closed’ community, restricted site access, and greater infection transmission prevention controls. Samples were analysed for norovirus (GI and GII), influenza virus (A and B), RSV (A and B) and SARS-CoV-2. In total, 93 samples were analysed for these 7 targets, with 27% of assays yielding detects (174 positive RT-qPCR results total, Fig 5). Positive detections were more frequent at the university (31% of assays) than the care home (22%), perhaps due to the university having a larger catchment population (cf. Fig 4) or successful infection prevention control measures at the care home. The distribution of positive assays between different viral targets was similar for the two sites (Fig F in S1 Text).

Norovirus comprised 55-59% of all positive assays. This is not surprising since norovirus is a gastro-intestinal pathogen known for strong faecal shedding profiles, potentially lasting more than a month [64], especially where the carriers have weaker immune systems [65]. Of the two norovirus genotypes monitored in this study, norovirus GII was detected more frequently than norovirus GI (GII: 71% of all university samples and 64% of care home samples, GI: 47% and 31% respectively) (Fig 5). This is also to be expected, since GII represents 70-80% of norovirus clinical outbreaks [45] and the prevalent GII.4 strain has particularly severe symptoms [66]. Greater overall loads of norovirus GII RNA were also detected in comparison with GI (Fig G in S1 Text). Whilst ammonia measurements suggested a greater human contribution to wastewater samples at the university than the care home (Fig Q in S1 Text), the comparable norovirus GII wastewater detection levels between the two sites may suggest vulnerable individuals shedding RNA in greater amounts at the care home.

Influenza A virus and SARS-CoV-2 were the next most prevalent pathogens, with similar detection rates between the two sites (16-17% and 11-15% of all positive assays). At the time of sampling, SARS-CoV-2 was at a temporary minimum, whilst the winter flu season had yet to begin. The frequent detection of SARS-CoV-2 and influenza A virus during times of low prevalence highlights the sensitivity of wastewater surveillance using RT-qPCR. RSV A was detected frequently at the university (27% of all samples) but much less so at the care home (11%). Influenza B virus and RSV B were rarely detected. For influenza virus, this is expected since influenza B is much less prevalent than influenza A [17]. However, RSV A and B genotypes are often detected in similar proportions to one another [17,67]. Overall, all pathogens except for influenza B virus were detected more frequently at the university than the care home. This is not surprising, since a larger population at the university provides a greater chance of finding at least one shedder (approximately 1000 people versus 190). Influenza B virus was an outlier, being more prevalent at the care home than the university (forming 5% versus 1% of all positive assays). This potentially indicates a localised outbreak.

#### 3.2.2. Norovirus time series.

**Fig 6:**
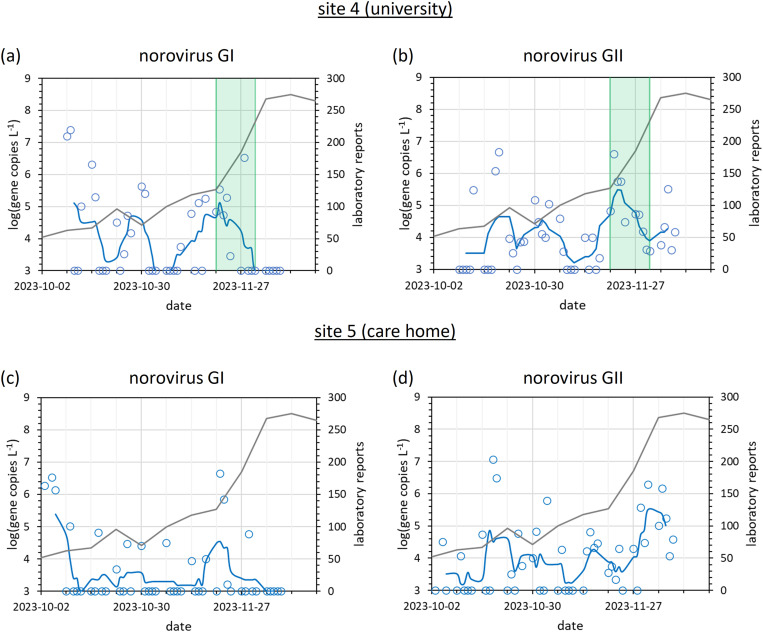
Timeline of norovirus GI and GII wastewater detections, alongside the number of weekly national surveillance laboratory reports at (a-b) site 4 (university), and (c-d) site 5 (care home) between October and December 2023. Daily wastewater RT-qPCR results are expressed as blue circles, the five-point moving average as a solid blue line (both on the left-hand axis), and national surveillance data as a solid grey line (right-hand axis). The green shaded region indicates the two weeks in which the university received a norovirus risk warning (on the basis of rising wastewater signals) and consequently implemented an information campaign and enhanced cleaning measures.

The norovirus time series is presented in Fig 6. At the university, both norovirus GI and GII were detected in three clusters (Fig 6A-B). Strong GI and GII detections were observed in the first and second weeks respectively (commencing 2023-10-09 and 2023-10-16). A second peak was then observed between 2023-10-23 and 2023-11-03. A third, but more consistent peak, with detections of GII on every day, and GI on 8 out of 10 days, was observed between 2023-11-13 and 2023-12-01. The initial detections might be linked to the start of term (2023-10-03) and community mixing. During the week commencing 2023-11-20, the university facilities management team were alerted to increases in wastewater norovirus detection (both frequency and concentration) and consequently implemented (a) more thorough cleaning, especially of touch points (door handles, bannisters etc), and (b) an information campaign (posters in every bathroom to warn of a rise in winter sickness and to recommend hand washing with soap). Enhanced cleaning can reduce microbial contamination of touch points (e.g., door handles and banisters) by 32% [68]. Both norovirus GI and GII wastewater signals declined over the next two weeks. Simultaneously however, many students also left campus for winter break, with the average daily headcount slowly declining from 1000 on week commencing 2023-11-20, to 600 on week commencing 2023-12-04. The decrease in wastewater norovirus signals is more dramatic than would be expected from a 40% decline in population alone (especially total suppression of the wastewater norovirus GI signal whilst national surveillance norovirus reports continued to increase, Fig 6A). Overall, we were not able to confirm whether the suppression of norovirus wastewater detections was due to changing catchment populations, intervention measures, or a combination of both.

The detection of norovirus was less consistent at site 5 (care home) than site 4 (Fig 6C-D). Beside initial detections in week commencing 2023-10-02, no significant GI peak was observed until 2023-11-20, with a peak lasting a few days only. Norovirus GII detections were more consistent, with a strong signal in week commencing 2023-10-16 followed by consistent detections at lower RNA concentrations until a significant increase began in week commencing 2023-11-27. Like the university, this final norovirus GII peak is likely linked to the start of the winter norovirus season, entering an exponential growth phase, with national surveillance showing the first winter norovirus peak plateau on week commencing 2023-12-12 [51]. Despite care home guidelines having a number of measures to prevent transmission of pathogens such as norovirus (e.g., PPE and good hygiene practice) [30] and the community being smaller and more isolated than at the university, norovirus detections were nearly as frequent, and just as strong, potentially due to vulnerable elderly residents exhibiting greater shedding loads and longer shedding profiles [47].

#### 3.2.3. Respiratory panel time series.

**Fig 7:**
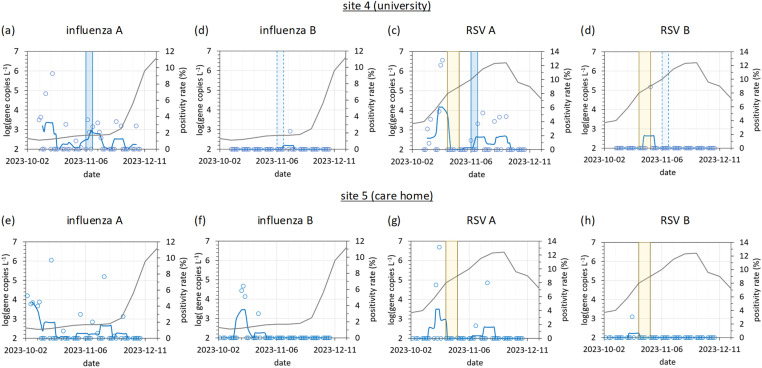
Timeline of the respiratory panel showing wastewater influenza A and B virus, RSV A and B detections, alongside national surveillance weekly positivity rates, at (a-d) site 4 (university) and (e-h) site 5 (care home). SARS-CoV-2 data is presented in Fig 1. Daily wastewater RT-qPCR results are expressed as blue circles, the five-point moving average as a solid blue line (both on the left-hand axis), and national surveillance data as a solid grey line (right-hand axis). The blue region indicates the week in which the university team reported one team member sick from “COVID-like symptoms” and another with a sore throat. The yellow region indicates the half-term school break. Reports of sickness amongst residents and staff at the care home were not available.

The time series of respiratory panel results are shown in Fig 7. At the university, influenza A virus was clustered around three moments in time. The first cluster of four positive detections occurred during week commencing 2023-10-09, one week after the start of term. The second cluster (six positive results) occurred during week commencing 2023-11-06, where staff reported one colleague off sick with flu-like symptoms and another with a sore throat (Fig 1A-D, blue shading). Influenza A virus, SARS-CoV-2 and RSV A were all detected during this week. Prior to the field study, the investigators expected that significant wastewater influenza virus detections would be observed towards the end of the study period, due to the onset of winter flu season. However, this was not observed with three detections only in the third cluster (week commencing 2023-11-20). This is in line with national surveillance data, which showed a delay to the flu season of four weeks versus the previous year, and the positivity rate only rising above 2% in the final week of field work. Influenza B virus was detected only once at the university (2023-11-14).

In contrast, a clustering of wastewater RSV A detections at the university was observed between 2023-11-06 and 2023-11-27, occurring at the same time as the annual peak in national RSV positivity rates (tests reaching 12.4% positivity). The start of these detections corresponds to the week that staff sickness was reported. Like influenza A virus, additional detections during the first week of the study period were also observed. RSV B was detected once only (2023-10-30).

At the care home, greater differences were observed between wastewater and national surveillance data, both for influenza virus and RSV. Influenza A virus detections were scattered throughout the study period, and like the university, a systematic rise in influenza A virus detections was not observed. However, a distinct influenza B virus peak was observed between 2023-10-16 and 2023-10-26, whilst national influenza positivity rates remained low. Influenza B virus detection in wastewater is typically rare and erratic [17,54] and this peak likely indicates a localised outbreak.

Unlike the university, there were only two detections of RSV A during the period of national surveillance maximum RSV positivity rates. This is perhaps due to effective infection prevention controls within the closed community. RSV B was detected once only.

Whilst the care home included a nursery within the sampled sewer catchment area, the university did not. However, a nursery was immediately adjacent to the sampled university building, and it is likely that children would use the café and canteen facilities. Young children experience particularly severe symptoms and significant shedding from RSV [35,69], and their contributions to the sampled wastewater effluent may account for some of the RSV A detections observed at the university when the national positivity rate was highest. We note that no RSV was detected at either site during primary school half-term breaks, where nursery attendance may have been lower (yellow shaded area in in Fig 7C-D,G-H).

### 3.3. Correlation and concordance between near-source and national-scale results

We considered several ways in which the end-user might interpret near-source wastewater data: (1) individual or (2) five-point moving averages considered in isolation to the rest of the time series, as well as changes in the time series using (3) day-to-day measurements, and (4) week-to-week aggregate measurements.

**Fig 8:**
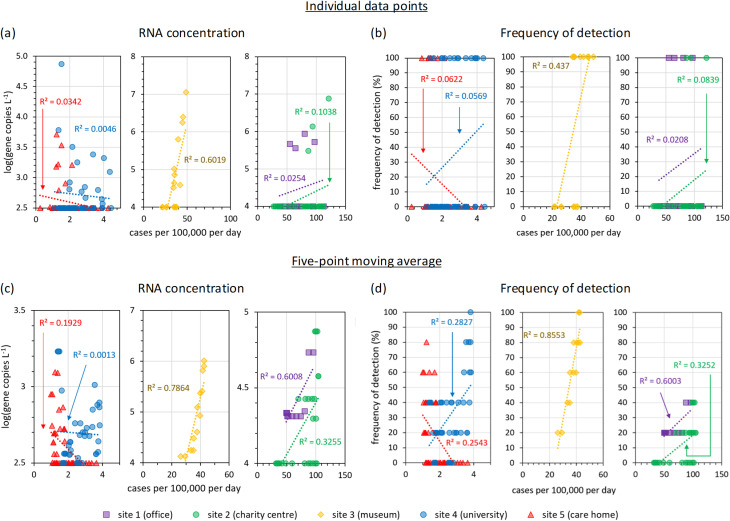
Correlation between near-source wastewater SARS-CoV-2 detections and national surveillance, using individual daily data points (top row) and a five-day moving average (bottom row). Moving averages were calculated for both wastewater data and case numbers, using five working days. For non-detects, the gene copy number was set equal to the limit of detection.

Firstly, we assessed the correlation between individual wastewater measurements and national surveillance case numbers. Individual, instantaneous wastewater measurements without time series contextualisation may have use cases where rapid responses to any single detection are required (e.g., pre-pandemic SARS-CoV-2 or bioterrorism agents [70]) or where guideline limits are used to determine risk levels (e.g., if future guidelines for norovirus were established given its short 1.2 days incubation period [42]). At sites 1-4, wastewater SARS-CoV-2 tended to be detected more frequently and in higher concentrations on days with higher reported case numbers (Fig 8A,B). However, the log-linear correlation between daily case numbers and wastewater SARS-CoV-2 RNA concentrations was poor, with R^2^ values below 0.11 at most sites. A negative correlation was observed at the care home. The exception was site 3, with an R^2^ value of 0.61. This site (the museum) had the largest catchment size, and its greater correlation agrees with the previous discussion (Fig 4).

Secondly, we assessed the correlation between the moving averages of daily wastewater measurements and national surveillance data. The variance between consecutive near-source wastewater RNA measurements at short timescales includes factors such as the stochastic behaviour of ‘super shedders’ who may have disproportionate contributions towards the RNA measured in near-source samples. Furthermore, the majority of our wastewater samples returned negative results for SARS-CoV-2 RNA (this agrees with previous near-source studies, where typically less than 50% of all assays yield positive results for SARS-CoV-2 RNA [26,29,48,54,71]). It is not surprising that Fig 8A yielded poor correlation coefficients. We therefore considered a five-point moving average to reduce the significance of unrepresentative samples. R^2^ values increased at all sites (Fig 8C,D) and this increased correlation is explained by time series smoothing, which reduces the number of ‘null’ results and provides broader wastewater peaks which overlap more with the reported case numbers. Moving averages also reduce the influence of weekday periodicity, which is stronger in the wastewater data set (Fig 3). The correlation coefficient between near-source wastewater results and national surveillance data continues to depend upon the sewer catchment size and COVID-19 prevalence (Fig K(a),(c) in S1 Text). The results indicate that besides (i) catchment size, (ii) viral prevalence, and (iii) how closed or open a community is, the aggregation of multiple results into a statistical average also increases the agreement between trends in near-source wastewater data and case numbers reported in the wider community. Since many near-source operational decisions will be made on day-by-day timescales, the aggregation of multiple samples into a moving average or other metric would be best achieved using higher frequency sampling (i.e., analysing multiple samples each day), which may be one motivation for the introduction of automated on-site detection [14].

Third, we assessed the concordance between day-by-day changes in wastewater and national surveillance results. When individual measurements are used to take mitigating actions, well-defined thresholds are necessary to map risk levels onto surveillance data [72]. However, guideline threshold values for wastewater pathogen nucleic acid concentrations do not yet exist [73,74] and it may be preferable to use previous data to contextualise the latest results. We subsequently considered whether rising or falling trends in near-source wastewater data correlate with national surveillance data. Firstly, considering the day-to-day change between two consecutive samples, concordance between near-source wastewater SARS-CoV-2 RNA and COVID-19 case numbers was poor-to-non-existent: the two parameters tracked one another only 50-67% of the time (Fig K(b) in S1 Text). Any concordance is likely due to strong weekday periodicity. Concordance at the largest catchment (site 3) significantly improved when replacing a single day’s measurements with a linear regression calculated across the latest five measurements (from 56% to 85%, Fig K(d) in S1 Text). The greatest concordance (89%) was achieved by including a threshold, to exclude days where wastewater SARS-CoV-2 measurements or COVID-19 case numbers changed by less than 10% (Fig L in S1 Text).

**Fig 9:**
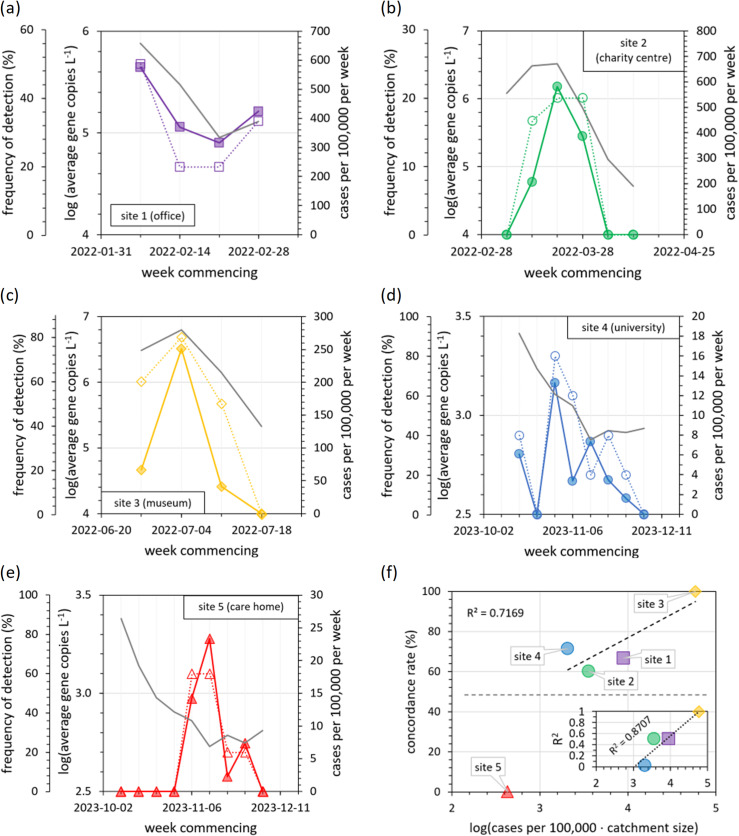
Weekly aggregates of near-source wastewater pathogen detections and concordance with national surveillance data. (a) to (e) Time series presented as the Monday-to-Friday average RNA concentrations (filled shapes with solid lines), detection frequency (open shapes with dashed lines), and COVID-19 case numbers (solid grey line, no shapes). (f) The influence of catchment size and viral prevalence on concordance rates between changes in weekly wastewater detection frequency and COVID-19 case numbers. The inset figure shows the goodness-of-fit (R^2^) calculated for correlations between the weekly wastewater detection frequency and COVID-19 cases. Correlation and concordance plots are provided in Supporting Information S1 Text.

Fourth, we assessed the concordance between week-by-week changes in wastewater and national surveillance results (both SARS-CoV-2 and norovirus GII). We considered an end-user consulting wastewater data less frequently, using weekly aggregates of average RNA concentration, detection frequency, and total case numbers. Qualitatively, SARS-CoV-2 wastewater data from sites 1-4 matches most key features of the national surveillance data (e.g., the timing of wastewater peaks, Fig 9A-E). Concordance is greater for week-by-week aggregates than day-by-day changes (for sites 1-4, concordance increased from 56-85% for day-by-day changes using a five-point linear regression to 67-100% for week-by-week changes, Fig 9F). Increased correlation and concordance between near-source wastewater and national surveillance measurements on week-by-week timescales indicates that the correlation seen in Fig 2 is not due to weekday periodicity alone (Fig 3), but it is also due to longer-term trends in increasing and decreasing SARS-CoV-2 prevalence. The care home shows no concordance between weekly wastewater SARS-CoV-2 data and national surveillance case numbers, further evidence that this small catchment with a closed community and greater infection prevention controls can be considered as a different environment versus the wider community (Fig 9E).

**Fig 10:**
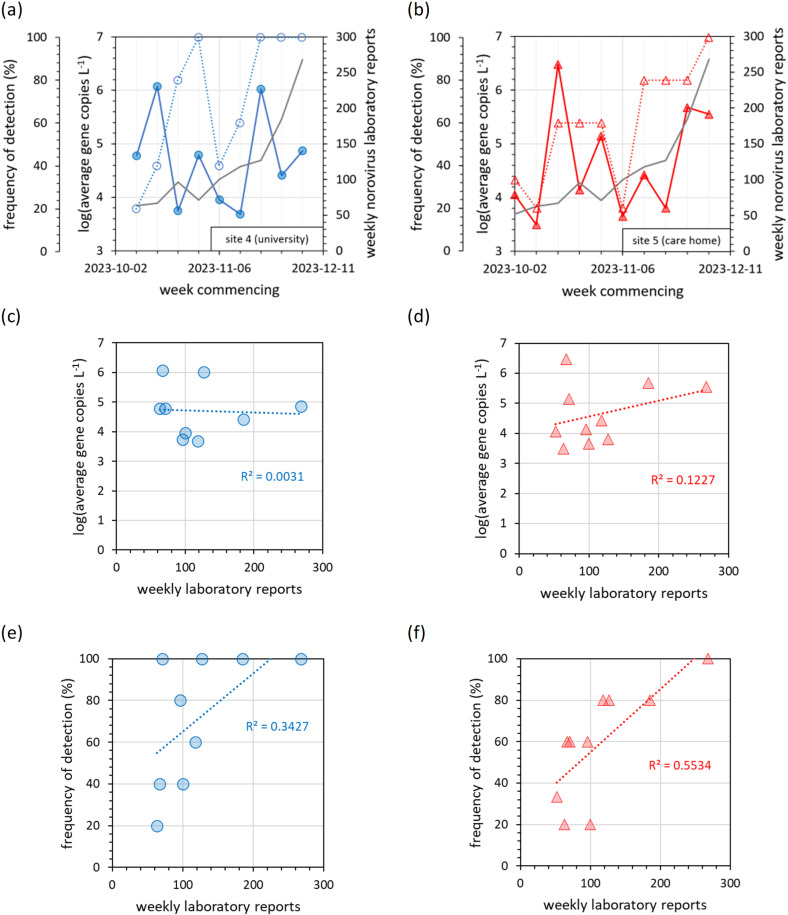
(a) and (b) Time series of weekly aggregates of norovirus GII RNA concentrations (filled shapes with solid lines), detection frequency (open shapes with dashed lines), and national surveillance laboratory report numbers (solid grey line, no shapes). Correlations of national surveillance data with wastewater RNA concentrations at (c) site 4 and (d) site 5, and with wastewater detection frequencies at (e) site 4 and (f) site 5. The correlation coefficient in (e) is limited by the natural plateau when detection frequency approaches 100%.

Box plots show a correlation between norovirus GII near-source wastewater detection and national surveillance data at both the university and care home (Fig H in S1 Text). However, correlation is not observed for any of the other pathogens, despite RSV A detections at the university clustering around the same time as peak national surveillance positivity rates (Fig 7C). Factors may include both the low prevalence of influenza virus and RSV during the study period, and the shorter faecal RNA shedding profiles and lower RNA loads of respiratory illnesses versus gastro-intestinal norovirus [35,47,64]. Correlation for norovirus GII but not for norovirus GI is explained by GII being the prevalent strain captured within the national surveillance reports (representing 70-80% of norovirus outbreaks [45]). Wastewater norovirus GII RNA concentrations showed significant week-to-week variability and no overall trend in the time series, whilst both wastewater detection frequency and national surveillance reports increased during the study period (Fig 10A,B). Consequently, national surveillance reports did not correlate to RNA concentrations (Fig 10C,D) but did correlate to wastewater detection frequencies (Fig 10E,F). There was no concordance in week-by-week changes (Fig P in S1 Text) due to the high week-to-week variability in wastewater metrics (Fig 10A,B).

Overall, there is evidence that the correlation and concordance between near-source wastewater surveillance and national surveillance data depend upon the combination of catchment size and viral prevalence. Negative correlation and zero concordance were observed for SARS-CoV-2 at the care home (site 5, Figs 8 and 9F). Here, the catchment size was small, the community was relatively sheltered from outsiders, and contemporary SARS-CoV-2 viral prevalence was low. Consequently, wastewater detections during November 2023 potentially indicate a localised outbreak. The university (site 4) shows the second weakest correlation and limited concordance. This is explained by low SARS-CoV-2 prevalence during the sampling period, despite a large catchment size. Greater correlation was observed at the office (site 1) and charity centre (site 2), where both sites were sampled during a time of high SARS-CoV-2 prevalence (though catchment sizes were small) (Fig 8). The greatest correlation and concordance was observed at the university (site 3), which was both sampled during high SARS-CoV-2 prevalence and featured the largest catchment population (Figs 8 and 9). Both SARS-CoV-2 at sites 1-3 and norovirus GII at site 4 showed correlation between near-source wastewater and national surveillance data, however only SARS-CoV-2 showed concordance (Fig 9F and Fig Q(d) in S1 Text). Potentially, SARS-CoV-2 signals consisted of small amounts of RNA shed by many individuals, creating a statistical smoothing effect not observed for norovirus GII, where a small number of individuals would have shed much greater amounts of RNA per infected individual (only around one in twenty people are infected with norovirus each year in the UK [75]). SARS-CoV-2 aggregate time series were similar whether using (a) RNA concentrations or (b) detection frequency and typically correlated to national surveillance data (Fig 9). However, for norovirus GII, only detection frequency correlated to national surveillance data; RNA concentrations did not (Fig 10). This further suggests that the norovirus GII signal is more heavily weighted than the SARS-CoV-2 signal by a minority of individuals shedding large but highly variable amounts of RNA.

## 4. Discussion

During this study, near-source wastewater surveillance was used to identify potential shortcomings in the LFD screening policy at an office, and actionability included scheduling enhanced cleaning and running information campaigns to highlight seasonal viral risk. An analysis of learnings from stakeholder discussions is provided in the Supporting Information (Table D in S1 Text).

Notable feedback included comments around baselining results and data confidence. The museum data user encountered challenges understanding the risk level associated with a wastewater SARS-CoV-2 peak during the BA.4/BA.5 outbreak because the peak occurred just two weeks into the field study. The data user was more familiar working to well-established threshold values (e.g., government regulated parameters for Legionella). However, with six weeks of baselining data, stakeholders at the university were confident to begin making decisions supported by wastewater surveillance data (enhanced cleaning and information campaigns). University stakeholders additionally highlighted the non-invasive qualities of the surveillance programme. Firstly, once sampling had begun, the investigators collected all samples and all data without requiring further action from university staff. Secondly, a discrete sampling location away from pedestrian walkways meant that there was no impact on day-to-day activities at the campus.

**Fig 11:**
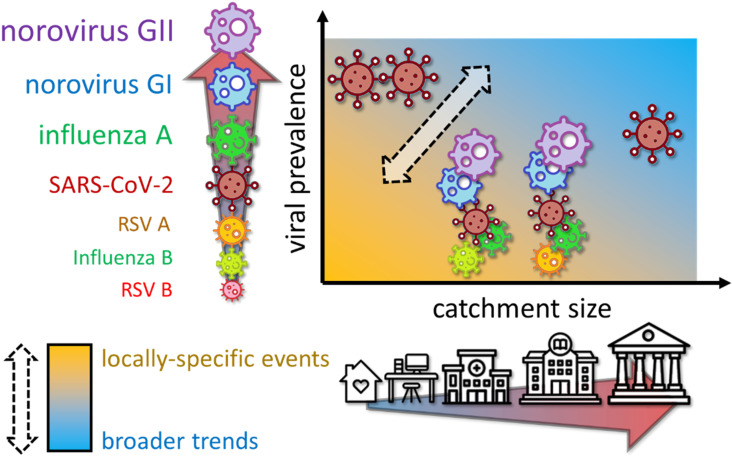
Qualitative summary of trends observed in this study, relating catchment size and viral prevalence to (a) the extent to which near-source wastewater surveillance revealed locally-specific events, and (b) the extent to which near-source wastewater surveillance matched and predicted national trends.

Our results indicate two broad use cases for near-source wastewater data. The first is around obtaining local insights. When catchment sizes are small or viral prevalence is low, near-source wastewater data reveals patterns not observed in the wider community and not captured by national surveillance programmes (Fig 11, lower left quadrant). Examples include identification of potential shortcomings in the LFD testing policy at the office, the correlation of respiratory pathogen detections with staff sickness at the university, and possible local presence of influenza B virus at a care home. In these scenarios, near-source wastewater surveillance might support an understanding of where local communities are vulnerable, and help evaluate the effectiveness of local interventions [26]. Sentinel wastewater monitoring provides a low-cost, non-invasive, continuous stream of background information, potentially identifying pathogens days before clinical results. This may prove invaluable when any local detection requires an intervention, e.g., bioterrorism threats [70], or where the symptoms of a particular outbreak are common across multiple pathogens, but each requires a different response (e.g., cleaning surfaces in response to norovirus versus improved food hygiene in response to *Campylobacter* spp.) [76].

The second use case is to provide an early warning of wider trends. When catchment sizes are large and viral prevalence is greater, near-source wastewater data begins to mirror macroscale national surveillance (Fig 11, upper right quadrant). In these scenarios, near-source wastewater monitoring might find applications ground-truthing anecdotal evidence, especially when wastewater data reporting is days or weeks faster than national surveillance. This would be similar to the municipal-scale wastewater early warning system, but with more confidence in geographical and demographic relevance. The main example in this study was a wastewater SARS-CoV-2 peak caused by the BA.4/BA.5 variant, detected at the museum nearly two weeks before city-wide results were available on the local government dashboard. Similarly, national surveillance norovirus reports were updated fortnightly, whilst updated wastewater results were available each day.

## 5. Conclusions

Near-source wastewater surveillance was conducted across five different sites for SARS-CoV-2 and two sites for norovirus, influenza virus and RSV. Our key findings are that (1) the product of catchment size and viral prevalence indicates whether a near-source catchment will reveal local events, or approximate the national surveillance picture. (2) Wastewater detections decreased in the order norovirus GII > norovirus GI > SARS-CoV-2 ≈ influenza A virus ≈ RSV A > influenza B virus ≈ RSV B. Norovirus was most prevalent likely due to high faecal shedding concentrations and long shedding profiles, and ratios between different genotypes followed clinically reported trends (GI versus GII, influenza A virus versus influenza B virus, RSV A versus RSV B). (3) SARS-CoV-2 wastewater detection frequency depended upon the product of catchment size and regional viral prevalence (following a log-linear relationship with R^2^ = 0.6914 to 0.9066). (4) Near-source wastewater SARS-CoV-2 data showed a strong weekday periodicity, stronger than periodicity in COVID-19 case numbers. The correlation between weekday-dependent wastewater detections and case numbers increases from R^2^ = 0.45 to 0.84-0.85 when modelling a variable delay lag time between the onset of faecal shedding and clinical diagnosis (average lag of 2.3 days).

Our results suggest beneficial use cases using near-source wastewater monitoring for both rare/high-risk pathogens in small communities, and endemic pathogens in larger communities. Further work should consider the use cases of multi-pathogen test panels in additional settings including the travel industry, e.g., cruise ships/ferry ports [71] and air planes/airports [77]. Additional research should consider the application of near-source wastewater monitoring to distinguish between outbreaks where pathogens present similar symptoms but require different mitigation efforts (e.g., the cleaning of touch points in response to norovirus versus improved food hygiene in response to *Campylobacter* spp.) [78].

## Supporting information

S1 Text Additional characterisation of catchments, pathogens, and national surveillance data; Table A: RT-qPCR reagents and controls; Fig A: Validation of centrifugal ultrafilter pre-concentration step; Table B: Influence of pasteurisation step on Ct values; Table C: RT-qPCR detection limits; Fig B: Modelling the weekday periodicity in reported COVID-19 case numbers using wastewater SARS-CoV-2 detection rates plus a variable lag time; Fig C-E: Correlation between wastewater SARS-CoV-2 detection frequency and the product of catchment size and COVID-19 case numbers; Fig F-H: Distribution of wastewater detections between different pathogens and box plot correlations to national surveillance data; Fig I-Q: Correlation and concordance of SARS-CoV-2 and norovirus wastewater detections with national surveillance data; Fig R-S: Characterisation of pH and NH3-N; Table D: Stakeholder feedback.
(PDF)

S1 Data Ct values and gene copies measured for each pathogen during field work, alongside national surveillance data; pH and NH3-N characterisation. (XLSX)
