# Supplementary material for: Near-source wastewater surveillance of SARS-CoV-2, norovirus, influenza virus and RSV across five different sites in the UK
Source: PLOS Glob Public Health. 2025 Apr 9;5(4):e0004397. doi: 10.1371/journal.pgph.0004397 (PMC11981152; doi:10.1371/journal.pgph.0004397)
Supplement: S1 Text — Additional characterisation of catchments, pathogens, and national surveillance data; Table A: RT-qPCR reagents and controls; Fig A: Validation of centrifugal ultrafilter pre-concentration step; Table B: Influence of pasteurisation step on Ct values; Table C: RT-qPCR detection limits; Fig B: Modelling the weekday periodicity in reported COVID-19 case numbers using wastewater SARS-CoV-2 detection rates plus a variable lag time; Fig C-E: Correlation between wastewater SARS-CoV-2 detection frequency and the product of catchment size and COVID-19 case numbers; Fig F-H: Distribution of wastewater detections between different pathogens and box plot correlations to national surveillance data; Fig I-Q: Correlation and concordance of SARS-CoV-2 and norovirus wastewater detections with national surveillance data; Fig R-S: Characterisation of pH and NH3-N; Table D: Stakeholder feedback. (PDF) [file pgph.0004397.s001.pdf]

Near-Source Wastewater Surveillance of SARS-CoV-2, Norovirus, Influenza Virus and RSV Across Five Different Sites in the UK

Jay C. Bullen[a]*, Mina Mohaghegh[a,]*, Fatima Tahir[a], Charlotte Hammer[b], Jacob Sims[a], Frederico Myers[a], Lucas Eisinger[a,c], Ali Reza Kasmati[a], Claire F. Trant[a,]*

[a] Untap Health, London, UK

[b] Cambridge Infectious Diseases, Department of Veterinary Medicine, University of Cambridge, Cambridge, UK

[c] Lifescience Dynamics, London, UK

*Corresponding authors: jay.bullen.phd@gmail.com (JCB); claire@untaphealth.com (CFT); mina@untaphealth.com (MM)

1. Table of Contents

1. Table of Contents 2
2. Additional Details on Wastewater Catchments and National Surveillance Regions 3
4. Description of Pathogens Investigated in This Study 4
5. RT-qPCR Reagents and Controls 5
6. Nucleic Acid Extraction Methods 5
7. Development and Validation of Ultrafiltration Wastewater Pre-Concentration Method 7
8. Influence of Pasteurisation Step on RT-qPCR C_t Values 8
9. Detection Limits 9
10. Investigation of differences between the weekday periodicity of wastewater SARS-CoV-2 detects and COVID-19 case numbers 10
11. Correlation Between Wastewater SARS-CoV-2 Detection Frequency and the Product of Catchment Size and COVID-19 Case Numbers 12
12. Distribution of Positive Detections Between Pathogens Monitored at the University and Care Home 16
13. Box Plots for Norovirus, Influenza A Virus, and RSV A 17
14. Daily Changes in SARS-CoV-2 18
15. Week-by-Week Correlation and Concordance in SARS-CoV-2 22
17. Week-by-Week Correlation and Concordance in Norovirus GII 25
18. Comparison of Correlation and Concordance Metrics 26
19. Chemical Characterisation (pH and NH_3) at Sites 4 and 5 28
20. Data Communication to End Users 30
21. Feedback from Stakeholders and Data Users 31
22. References 33

2. Additional Details on Wastewater Catchments and National Surveillance Regions

Wastewater sampling strategy: At each site, autosamplers were installed to capture the greatest possible proportion of people using the site (i.e. as many toilets as possible) to provide the greatest chance of capturing locally observed events such as staff sickness, and such that headcount or other occupancy data would be relevant to the wastewater data set. Other considerations in autosampler location included accessibility and ensuring that traffic and pedestrians weren't obstructed. Autosampler sampling frequency was increased as much as battery life would allow (with batteries replaced daily). The start and end times for daily composite sampling were chosen to provide approximately one hour headroom before and after sites became meaningfully occupied, for instance one hour before site 1 (office) opened and one hour after it closed.

Site 2 (charity centre): Staff members used a non-contact infrared thermometer to measure the forehead temperature of all visitors. The site had two restrooms, one for men and one for women. The effluent from these restrooms met off-site, where daily access was not viable, and consequently only the women's restrooms were sampled (representing the majority of people on site). The charity centre was sampled from 10am onwards, as this is when staff first arrived and unlocked the site.

Site 3 (museum): The restaurant served 150 covers per day. Composite wastewater samples were collected from a three-story stack, with effluent captured from approximately one third of the restrooms on site. Sampling was timed to start shortly before, and finish shortly after, peak footfall (including office workers), on the basis of site occupancy data.

Site 4 (university): The headcount was performed 6 times per day, at approximately two-hour intervals. Data from the on-site canteen provided a similar estimate, with ~650 table covers and 1200 transactions per day consistently across the study period. The wastewater sampling point covered the entire co-working building. The headcount used for calculations was the daily sum of headcount data, averaged over the entire course of the field work study. Sampling was timed to start before and finish after peak footfall (including office workers), on the basis of headcount data.

Site 5 (charity centre): Sampling continued until 11pm, since residents were still active until this time.

National surveillance catchments: The COVID-19 national surveillance catchment areas were Southampton (250,000 people, representing the community surrounding site 1), London (8.9 million, representing sites 2 and 3), the East of England (9.3 million, representing site 2), and the South East of England (6.2 million, representing site 5).

National surveillance data on COVID-19 case numbers, publicly available from the UK government, included 'Pillar 1' and 'Pillar 2'. Pillar 1 cases are those identified through testing within a healthcare setting, whilst Pillar 2 cases are identified through community testing, including home testing [1]. The majority of reported tests are from Pillar 2 (97% as of April 2022), of which 71% are RT-qPCR tests (or LFD followed by RT-qPCR), whilst only 29% of recorded cases are attributed to LFD results only [2]. Case numbers are reported from the date that the sample specimen was collected, and not from the date of symptom onset. Daily SARS-CoV-2 case data was collected from the UK government coronavirus dashboard [3] and the Mayor of London's London Datastore [4].

4. Description of Pathogens Investigated in This Study

SARS-CoV-2: COVID-19 is a respiratory illness, with the most common symptoms being fever and coughing [5]. Studies report up to 79% of cases being asymptomatic [6] and 30% [7] to 50% [8] of secondary transmission occurring before the onset of symptoms. Superspreaders, with viral loads up to fifty times higher other infected individuals, comprise 15 to 19% of cases, but cause up to 80% of secondary transmission [8]. The incubation period between SARS-CoV-2 exposure and the onset of symptoms has decreased as new variants have emerged, e.g. 4.2-4.9 days for Alpha [9,10], 3.7-4.4 days for Delta [8,9,11], 3-3.7 days for Omicron BA.1 [8,9,11], 3.5 days for Omicron BA.2 [9], and 2.4-2.6 days for BA.5 [12,13]. The peak in viral load (including the RNA that is measured during a PCR diagnostic test) typically lags behind the onset of symptoms for 1 [14], 2 [15] or 3 [16,17] days for Omicron BA.1 and 2 days for BA.2 [18]. Studies have reported that lateral flow device (LFD) tests only offer a 70-80% chance of a true positive when taken on the same day as the onset of symptoms [19]. Tests are rarely administered daily, and studies have reported a median two day lag between the onset of symptoms and positive LFD results [8,20]. Elsewhere, studies highlight the additional delay between the onset of symptoms and diagnosis due to human factors, e.g. individuals delaying the seeking out and conducting of a diagnostic test [21]. COVID-19 diagnostic testing underreports true case numbers: It's estimated that only 20-40% of UK COVID-19 cases are identified correctly by positive test results, and that Omicron BA.2 cases are correctly identified 2-3 times less successfully than Alpha, Delta and Omicron BA.1 cases [1]. In the analysis of stool samples, SARS-CoV-2 RNA can be detected in 25% [22] to 44% [23] to 62% [24] of COVID-19 patients. Faecal shedding measurements in 2020 suggested a peak after 6 days [25], and RNA detection can persist for 11 days [22]. SARS-CoV-2 RNA wastewater shedding rates increased during the transition from Alpha and Delta variants and decreased with the arrival of Omicron [26].

Influenza virus: Seasonal influenza contributes significantly to morbidity, mortality worldwide [27]. Annually, in the UK, approximately 2.4% of children below 5 years of age and 1.3% of seniors above 74 years of age consult their GP for an episode of influenza [28]. This is estimated to correspond to an average of 4.8 million lost working days per year attributed to influenza [29]. Influenza is highly contagious with an incubation period between one and three days [30] and an estimated R0 of 1.28 [31]. Similar to contemporary SARS-CoV-2 variants, influenza A viral loads peak 2 days after infection, with a mean viral shedding duration of 4.8 days and undetectable results after 8 days [32]. Wastewater signals might last as little as a couple of days [33]. Wastewater detection of influenza A virus tracked incidence rates at a US university with 10,000 individuals in the sewershed [33].

RSV: Human respiratory syncytial virus (RSV) is one of the most common causes of acute lower respiratory tract infection (LRTI) in young children and older adults [32]. In 2019, RSV affected approximately 33 million children worldwide [34]. In the UK, RSV accounts for morbidity and mortality predominantly of young children and the elderly with a total of nearly 500,000 GP appointments [35] and 8,482 deaths [36] per season associated with RSV and based on data from the US, the R0 of RSV is estimated to be 3.0 [37]. RSV viral loads peak 5.4 days after infection [32].

Norovirus: Norovirus is one of the most common causes of gastro-intestinal disease, with nearly 700 million cases per year globally [38]. R0 estimations for norovirus vary with a marked difference between population-wide estimates (approximately 2) and outbreak estimates (up to 7) [39]. Norovirus has longer shedding profiles than the respiratory illnesses discussed in this study, with shedding lasting for 7 to 12 days [40].

5. RT-qPCR Reagents and Controls

Table A: Table of positive control standards and RT-qPCR assays used in this work. Positive RNA controls were obtained from ATCC whilst RT-qPCR assays were obtained from ThermoFisher.

Name of the strain	Application	Concentration	Product code
Norovirus GI	Standard RNA, Positive control	10^5 gene copy/ µL	ATCC-VR-3234SD
Norovirus GII	Standard RNA, Positive control	10^5 gene copy/ µL	ATCC-VR-3235SD
Influenza A H3N2	Standard RNA, Positive control	10^5 gene copy/ µL	ATCC-VR-1882DQ
Influenza B (Florida/4/2006)	Standard RNA, Positive control	10^5 gene copy/ µL	ATCC-VR-1804DQ
Respiratory syncytial virus A (RSV A)	Standard RNA, Positive control	10^5 gene copy/ µL	ATCC-VR-1540DQ
Respiratory syncytial virus B (RSV B), strain 9320	Standard RNA, Positive control	10^5 gene copy/ µL	ATCC-VR-955DQ
SARS-CoV-2 (ORF, E and N genes)	Standard RNA, Positive control	10^5 gene copy/ µL	ATCC-VR-3276SD
SARS-CoV-2 (E and N2 genes)	cDNA, positive control	$2*10^3$ gene copy/ reaction	LuminUltra
Taqman Norovirus GI	Primers, qPCR amplification	20x	ThermoFisher Vi07922261_po
Taqman Norovirus GII	Primers, qPCR amplification	20x	ThermoFisher Vi07922262_po
Taqman Influenza A	Primers, qPCR amplification	20x	ThermoFisher Vi99990011_po
Taqman Influenza B	Primers, qPCR amplification	20x	ThermoFisher Vi99990012_po
Taqman RSV A	Primers, qPCR amplification	20x	ThermoFisher Vi99990014_po
Taqman RSV B	Primers, qPCR amplification	20x	ThermoFisher Vi99990015_po

6. Nucleic Acid Extraction Methods

LuminUltra method: Briefly, 1 mL of wastewater was mixed with 5 mL of lysis buffer containing proteinase K and incubated for 10 minutes at room temperature with occasional shaking. The sample-lysis buffer mixture was combined with 2 mL of ethanol, followed by the addition of 40 µL of magnetic beads to the solution. The mixture was then incubated at room temperature for 10 minutes to facilitate the adsorption of RNA onto the surface of the beads. After 10 minutes the supernatant was decanted using a magnetic rack to retain the beads. Next, 3 mL of wash buffer 1 was added to the beads and the supernatant was discarded using a magnetic rack. Then the magnetic beads were washed with 1 mL wash buffer 2 to ensure all the contaminants such as proteins were removed. The washed magnetic beads were then left to dry by incubating at room temperature for 15 minutes. After drying the magnetic beads under ambient conditions, the beads were rehydrated in 100 µL RNase-free water and the nucleic acids eluted through desorption at 65 °C for 3 minutes.

Amicon and Qiagen method: This method was developed to concentrate nucleic acid and improve the efficiency of the extraction process. Briefly, 30 mL wastewater sample aliquots were first pre-heated at 65 °C for 30 minutes, aiming to obstruct the outer capsid membrane of viruses, increasing the likelihood of nucleic acid release. Pasteurisation has resulted in stronger wastewater SARS-CoV-2

RT-qPCR detection in some studies [41], and weaker detection in others [42]. In our current study, pre-heating at 65°C for 30 minutes typically had a small, but potentially insignificant, improvement in the measured C_t value (by 0.44 cycles). The samples were centrifuged at 8194 rpm for 30 minutes to remove debris (centrifuge: Eppendorf 5430R and rotor: Eppendorf F-35-6-30). The pellet was discarded, and the supernatant was then loaded in aliquots into a Millipore Amicon centrifugal ultrafilter unit (100 kDa, 15 mL capacity). Ultrafiltration was conducted through centrifugation at 8194 rpm until the initial volume of 30 mL was reduced to 300 µL of retentate. The retentate was resuspended by pipetting and added to 1 mL of pre-heated PM1 buffer at 55 °C. The mixture was centrifuged at 11195 rpm for 1 minute, the pellet was discarded, and the supernatant was mixed with 200 µL of IRS buffer and incubated for 5 minutes at 4°C . The mixture was then centrifuged at 11195 rpm for 1 minute, the pellet discarded, and the supernatant mixed with 650 µL of PM3 and 650 µL of PM4 buffers. The sample was mixed gently before loading into RNeasy spin column. Loaded sample was centrifuged at 11195 rpm, the flow-through was discarded and the spin column was washed using 650 µL PM5 and centrifuged twice to remove potential remaining PM5 solution. 50 µL of a mixture of DNase I and digestive buffer was added to the centre of the spin column and incubated at room temperature for 15 minutes. The spin column was then washed with 400 µL PM7, 650 µL PM4 and 650 µL PM5 buffers respectively. Finally, RNA was eluted in 150 µL nuclease-free water. Eluted nucleic acids were analysed immediately or stored at -80 °C for up to two days prior to analysis.

7. Development and Validation of Ultrafiltration Wastewater Pre-Concentration Method

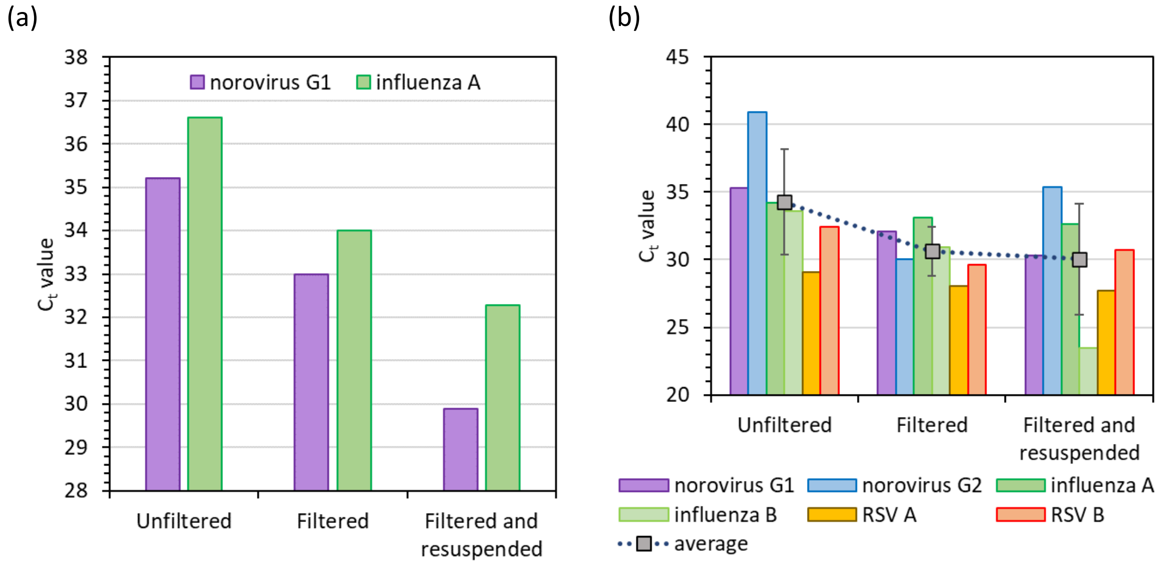

Fig A: Improved RT-qPCR detection sensitivity (and detection limits) using a centrifugal ultrafilter pre-concentration step. (a) data collected using Stratagene MX3000p qPCR instrument and 20 µL reaction volumes, and (b) data collected using TechnePrime 48 Pro qPCR instrument and 10 µL reaction volumes.

During 2022 field work, SARS-CoV-2 was detected using a magnetic bead-based method to extract RNA from wastewater samples. For the detection of seven target pathogens during 2023 field work, the RNA extraction method was replaced with a combination of Amicon centrifugal ultrafiltration, as a concentration step, and Qiagen PowerWater RNA extraction using silica minispin columns. The methodology change was due to challenges obtaining consistent RT-qPCR calibration curves when using the new RT-qPCR assays for norovirus, influenza virus, and RSV targets with the extracted RNA from the magnetic bead method (using an ATCC RNA standard dilution series).

Norovirus GI and influenza A RNA standards (ATCC) were spiked into deionised water. Samples were extracted using the Qiagen PowerWater RNA kit and analysed using RT-qPCR mastermix as described in the main text. C_t decreases of 2.2 and 2.6 for norovirus GI and influenza A virus respectively, when the sample was processed using an Amicon centrifugal ultrafilter prior to nucleic acid extraction (equivalent to concentration factors of ~4.6 and ~6.1). This increased to C_t decreases of 5.3 and 4.3 for norovirus GI and influenza A virus respectively, when the centrifugal ultrafilter's retentate was aspirated by pipette, to loosen the filter cake and resuspend solids. This is equivalent to enrichment factors of ~40x and ~20x respectively. Similar improvements were observed when a larger array of targets were spiked into deionised water, and extracted nucleic acids analysed using a TechnePrime 48 Pro qPCR instrument.

8. Influence of Pasteurisation Step on RT-qPCR C_t Values

Table B: The influence of a pasteurisation step on C_t values. The nine samples were real wastewater samples (from sites 4 and 5). The samples were split into two aliquots, of which one was subjected to a heat pre-treatment at 65 °C for 30 minutes. Both samples with and without the heat pre-treatment were then subjected to centrifugal ultrafiltration, RNA extraction, and RT-qPCR analysis.

Sample	Norovirus GI		Norovirus GII		Influenza A		Influenza B		RSV A		RSV B		SARS-CoV-2	
	no heating	with heating	no heating	with heating	no heating	with heating	no heating	with heating	no heating	with heating	no heating	with heating	no heating	with heating
1	-	-	38.36	36.89	33.65	35.89	-	-	-	-	-	-	-	-
2	-	-	-	-	37.0	39.0	-	-	-	-	-	-	-	-
3	38.0	-	-	-	-	36.01	-	-	36	-	-	-	-	-
4	36.3	34.68	36.1	-	35.35	-	-	-	34.35	35.85	-	-	-	-
5	-	-	-	37.0	-	38.0	-	-	-	-	-	-	-	37.66
6	-	-	-	-	-	-	-	-	-	-	-	-	-	-
7	36.6	-	-	-	-	37.1	-	-	38.0	-	-	-	-	-
8	-	38.9	-	-	39.16	-	-	-	-	-	-	-	-	-
9	-	-	35.1	35.4	-	-	-	-	-	-	-	-	-	-

Samples subjected to pre-heating at 65°C for 30 minutes were compared with untreated samples. There were marginally fewer detections when samples were pre-heated (12 versus 13 without heating), however the average C_t values was reduced by 0.44 cycles. The significance of these differences was not assessed using statistical means.

9. Detection Limits

The assay limit of detection (ALOD) was calculated by determining the C_t value at which there was a 95% probability of detection [43] [44] using the Thermofisher TaqMan RT-qPCR assays applied to a dilution series of ATCC RNA standards. 95% probability of detection occurred at approximately C_t 36.9 and corresponds to detection limits between 4 and 17 gene copies (gc) per reaction volume for the various pathogen targets. The sample limit of detection (SLOD) was obtained by normalising the ALOD to the volume of template RNA (10 µL) and then dividing by a concentration factor of 200, to represent the volume reduction during ultrafiltration and minispin column RNA extraction [45]. Final detection limits ranged between 2×10^3 and 9×10^3 gc per litre of wastewater (Table).

When using the magnetic bead-based extraction method, detection limits for SARS-CoV-2 were approximately 5.0×10^4 gc L^{-1} for N2/E genes [44] decreasing to 1.3×10^4 gc L^{-1} for the N1 assay. Detection limits improved by an order of magnitude when changing to a combination of centrifugal ultrafiltration and a minispin column-based extraction to approximately 2.6×10^3 gc L^{-1} whilst continuing to use the N1 assay.

Detection limits were similar to previous wastewater studies, lying between 1×10^3 and 1×10^4 gc L^{-1} (Table C) [45] [46] [47] [48] [49] [50]. For our time series analysis, we included positive detections below the 95% detection limit. Unlike most methods in analytical chemistry, PCR detections below the LoD are often regarded as true positives, as negative controls are expected to provide zero signal response and zero noise [43]. The logistic regression model used to determine the 95% ALOD also indicated that detections at cycle numbers 38 and 39 would be reproducible in 57% and 25% of repeats. We consequently included positive detections within our time series analysis, providing they obeyed all three criteria for environmental monitoring: C_t values were no greater than 40, qPCR curves were regular in shape, and no signal was observed in the no template negative controls [43].

Table C: Detection limits calculated for various wastewater assays.

Target	SARS-CoV-2		Norovirus GI	Norovirus GII	Influenza A virus	Influenza B virus	RSV A	RSV B
Extraction	Magnetic bead	Ultrafiltration and minispin column						
Assay	LuminUltra		Thermofisher					
PCR efficiency (%)	86	-	105	89	92	107	115	122
Assay limit of detection (ALOD) (gc)	10.5 (N2, E) 3 (N1)	3	17	6.3	4.5	3.8	4.2	15
Sample limit of detection (SLOD) (gc L^{-1})	5.0×10^4 (N2, E) 1.3×10^4 (N1)	2.6×10^3	8.6×10^3	3.2×10^3	2.3×10^3	1.9×10^3	2.1×10^3	7.7×10^3
Literature detection limits (gc L^{-1})	1×10^3, 2×10^3 [45]<br>1.3×10^3 [50]<br>6.7×10^3 [49]<br>1.9×10^4 to 2.9×10^4 [48]<br>5.0×10^4 [44]		4.5×10^3 [46]<br>Typically 2.2×10^3 to 3.2×10^4 [47]		6.0×10^3 [48]		7.3×10^3 [48]	

10. Investigation of differences between the weekday periodicity of wastewater SARS-CoV-2 detects and COVID-19 case numbers

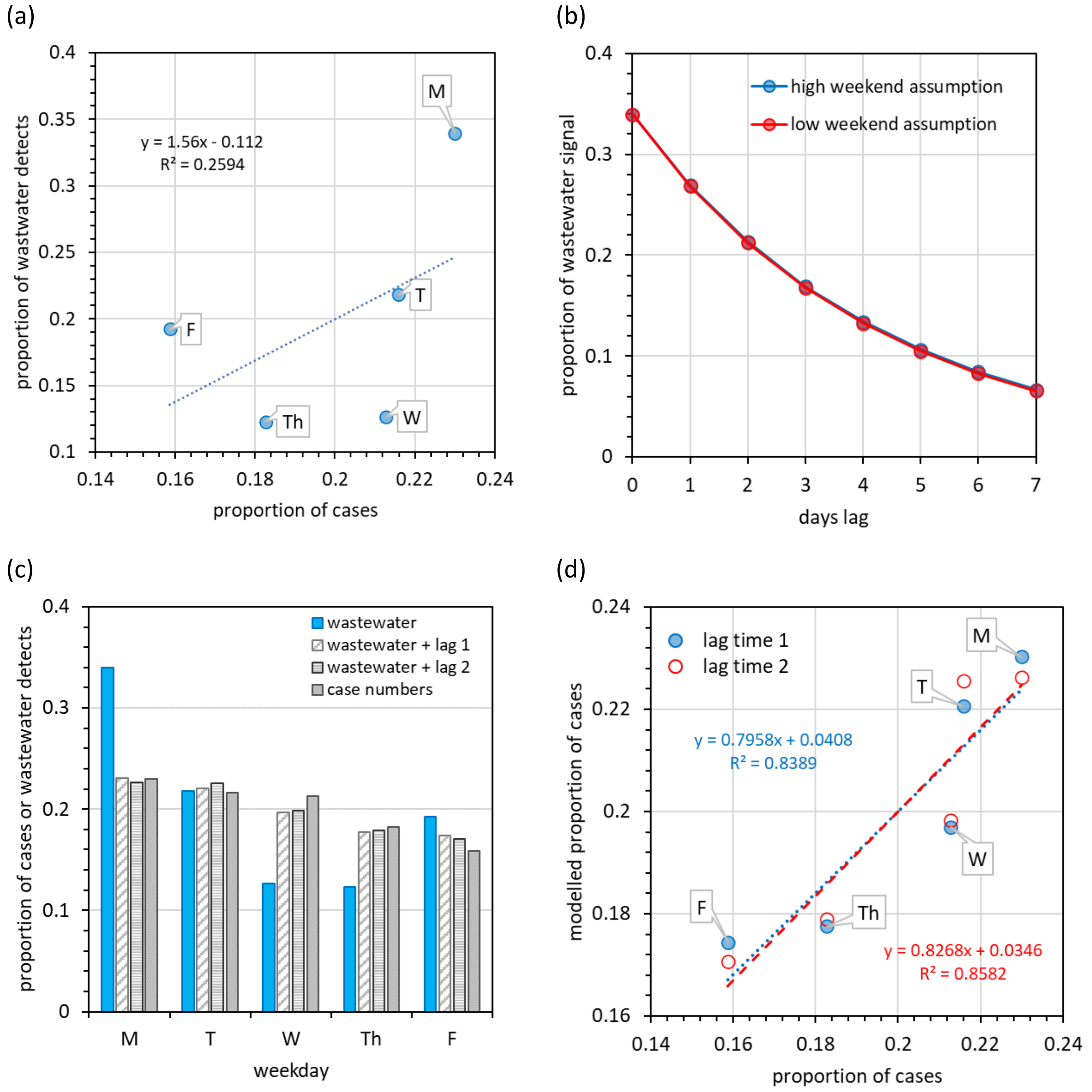

Fig B: Modelling the weekday-dependence of reported COVID-19 case numbers using the weekday-dependence of wastewater SARS-CoV-2 detection rates plus a lag time, where the lag time between SARS-CoV-2 shedding into wastewater and COVID-19 diagnosis varies according to an exponential decay distribution. (a) Limited correlation in the weekday-dependence of reported COVID-19 case numbers and wastewater SARS-CoV-2 detection rates without modelling a variable diagnosis lag period. (b) The exponential decay that is used to model the weekday-dependence of COVID-19 case numbers using the weekday-dependence of SARS-CoV-2 wastewater detection rates. The exponential decay factor was optimised to provide the best fit between the modelled weekday-dependence of COVID-19 case numbers and the weekday-dependence of COVID-19 case numbers reported in the national surveillance data set. The 'high weekend assumption' assumes wastewater detection rates on Saturday and Sunday are the same as detection rates on Monday (the weekly maximum), whilst the 'low weekend assumption' assumes that wastewater detection rates on Saturday and Sunday are the same as on Friday. (c) A comparison of weekday-dependence of wastewater SARS-CoV-2 detection rates (blue), reported COVID-19 case numbers (solid grey) and the COVID-19 case numbers using wastewater input data (grey patterned bars). 'Lag 1' and 'lag 2' refer to models using the exponential decay profiles from the 'high' and 'low' weekend assumptions in figure panel (b) respectively. (d) Improvements in the correlation between the weekday-dependence of reported COVID-19 cases (x-axis) and the weekday-dependence predicted by modelling wastewater data (y-axis).

To explain how wastewater SARS-CoV-2 detection rates exhibit greater week-day dependence than COVID-19 case numbers, we explored the possible lag times between viral shedding and diagnostic testing. Whilst SARS-CoV-2 RNA may be shed by an individual for days or weeks, an individual may only report one single date for a positive test result. Given that there is a wide variability in the delay between COVID-19 exposure, the onset of symptoms, viral titre peaks, and positive lateral flow test detections, we considered the case that the weekday effect might be real (rather than due to reporting biases), and that SARS-CoV-2 wastewater detection rates might show a greater week-day dependence than COVID-19 case numbers due to a greater variability in lag time between exposure and diagnosis than the variability between exposure and viral shedding. With the assumption that reported cases lag behind wastewater shedding by 0 to 7 days, and the assumption that the distribution of the number of days lag time can be approximated using an exponential decay, we optimised the exponential decay factor to fit the distribution of case numbers across each weekday, using the weekday-dependence of wastewater detections as input data (Fig B). The goodness-of-fit R^2 parameter between wastewater data and case number weekday distributions increases from 0.45 to 0.84-0.86 when the exponential decay lag factor between viral wastewater shedding and diagnostic testing is considered. The exponential decay factor of 0.21 gives an average lag time of 2.3 days. This is not dissimilar from the 1-2 day lag time between symptoms presenting, or PCR diagnostics giving a positive result, and a lateral flow device giving a positive result [8] [51] [52].

11. Correlation Between Wastewater SARS-CoV-2 Detection Frequency and the Product of Catchment Size and COVID-19 Case Numbers

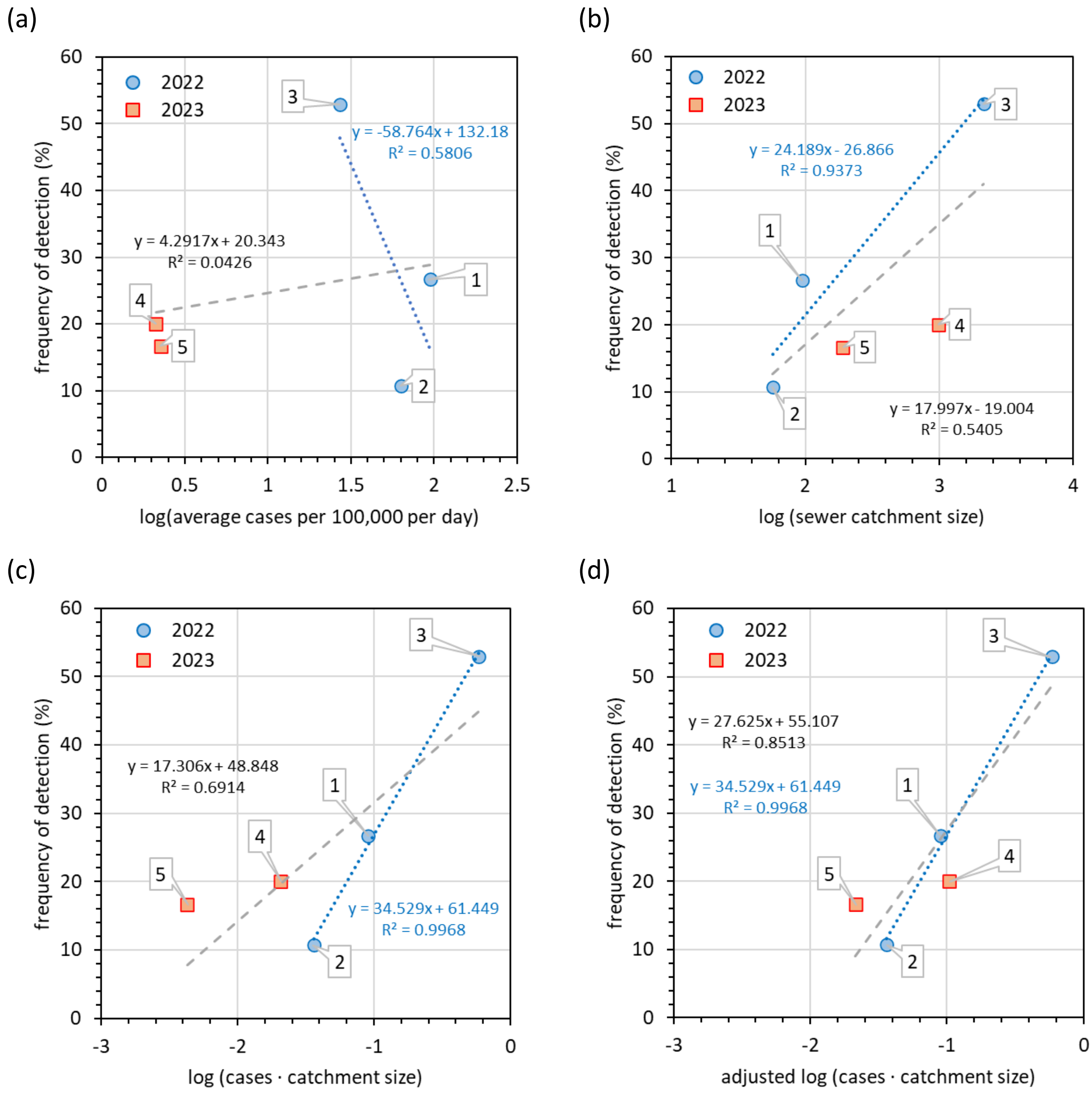

Fig C: Correlation between the frequency of wastewater SARS-CoV-2 detection and an aggregate of the sewershed catchment size and COVID-19 case numbers. In the analysis of linear regressions, the logarithm of COVID-19 case numbers and sewershed catchment size was used, on the basis that this varied by two orders of magnitude across the 5 sites surveyed, whilst frequency of detection was maintained on a linear scale, given a factor of four difference only between the greatest and smallest detection frequencies. (a) No correlation is observed between the frequency of wastewater detection and COVID-19 case numbers, when comparing different sites. (b) Correlation between the frequency of wastewater detection and the sewer catchment size. (c) Improved correlation between the frequency of wastewater detection and an aggregate of sewer catchment size and COVID-19 case numbers (new cases per capita). (d) Further improvements to the correlation when COVID-19 case numbers when including a normalisation factor to reflect the decrease in COVID-19 diagnostic testing rates between 2022 and 2023. Here, case numbers from 2023 were inflated by a factor of five. Grey dashed lines indicate the linear regression between all data points (2022 and 2023) whilst blue dotted lines indicate the linear regression for 2022 sites only. Data callout boxes provide the ID number of each site.

The main text demonstrates the correlation between wastewater SARS-CoV-2 detection and COVID-19 case numbers when assessing sites 1-4 individually (e.g. main text Fig 2). However, when comparing sites with one another, there is no correlation between the frequency of wastewater

detection and case numbers, Fig C(a). The sites differed in catchment size, and in the months and locations at which they were sampled, factors which prevent a correlation between unadjusted COVID-19 case numbers and wastewater detection frequency. In contrast, a positive correlation is observed between the sewershed catchment size and the frequency of wastewater detection (Fig C(b), R^2 = 0.5405 for all five sites and 0.9373 for sites in 2022 only). This reflects the increased probability of detecting a shedder within a larger population size. This correlation is improved when the catchment size is multiplied by the COVID-19 case numbers (Fig C(c), R^2 = 0.6914 for all five sites and 0.9968 for sites in 2022 only). The correlation is further improved when multiplying COVID-19 case numbers in 2023 by a factor of five, Fig C(c). This represents the decrease in individuals intending to use LFD tests from 40-60% in January 2022 to 10% at the beginning of 2023 [53] as discussed in the main text. The resulting correlation coefficient is R^2 = 0.8513 for all five sites and 0.9968 for sites tested in 2022 only.

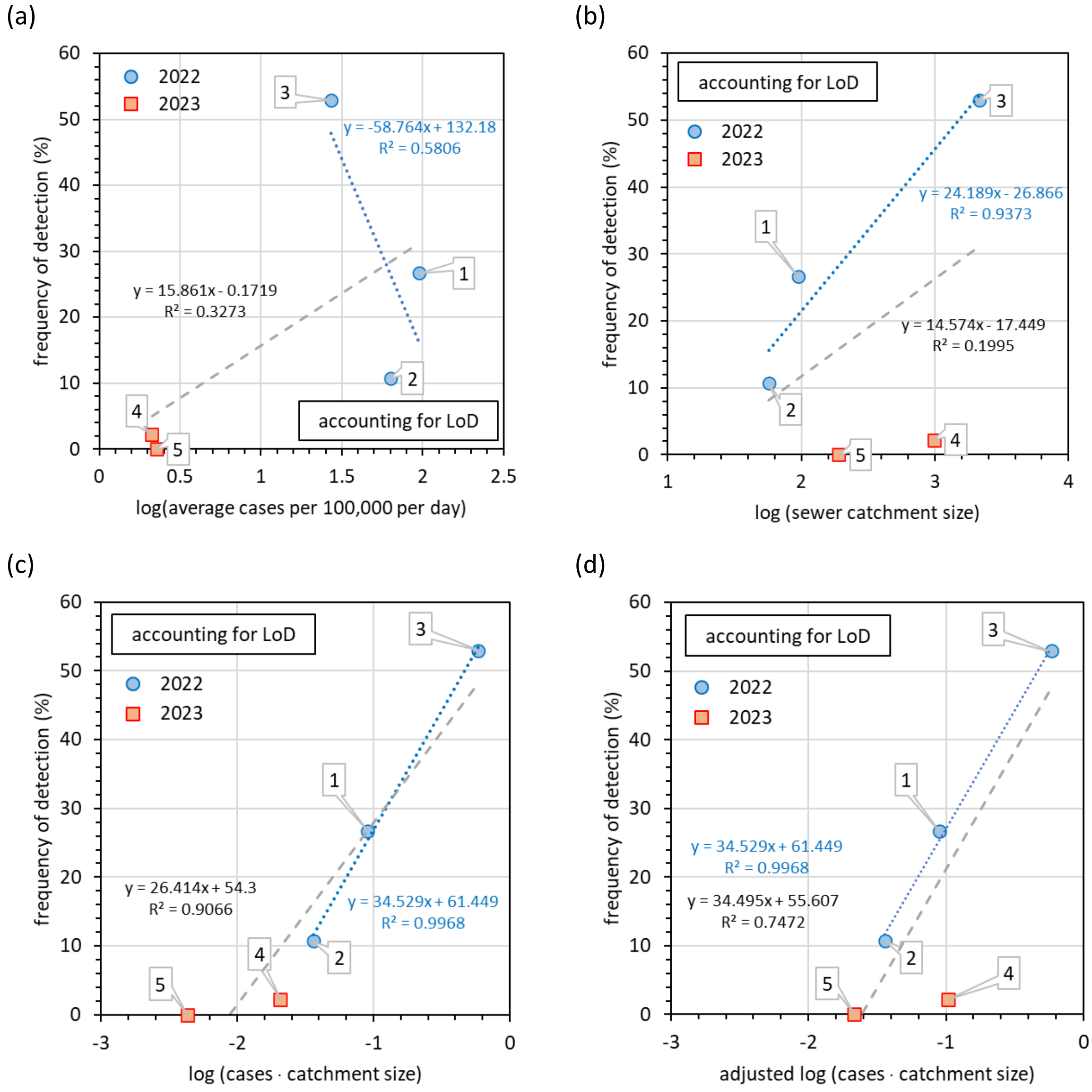

Fig D: Accounting for improvements in detection limits between 2022 and 2023 field work sites. Correlations between the frequency of wastewater detection and (a) the average number of new cases per 100,000 people per day for each region during each study period, (b) the number of people within each sewer catchment, (c) the logarithm of the product of the average number of new cases per capita per day multiplied by the number of people within each sewer catchment, (d) as per previous figure panel, but with 2023 case rates multiplied by a factor of 5 to account for variable LFD testing rates. To

account for differences in detection limits, any positive detections from sites 3-5 below the original detection limit at sites 1-2 were re-designated as non-detects. Data callout boxes provide the ID number of each site.

As wastewater RNA detection methods were improved during this study, detection limits also improved, increasing the probability of detecting SARS-CoV-2 RNA at low concentrations. Detection limits improved by over one order of magnitude when the original magnetic bead-based extraction method was replaced with a combination of centrifugal ultrafiltration and minispin column extraction (and to a lesser extent detection limits improved when upgrading the RT-qPCR assay). To assess the impact of changing detection limits on our analysis, we discounted any wastewater detections at sites 3-5 where the measured RNA concentration (gc L^{-1}) was less that the working detection limit at sites 1 and 2 (Fig D). We observe that there is still no correlation between SARS-CoV-2 detection frequency and the average number of new cases, Fig D(a). The correlation between detection frequency and the sewer catchment size is worse, with R^2 decreasing from 0.5404 to 0.1995 in Fig C(b) versus Fig D(b). However, the correlation between detection frequency and the product of case numbers with sewer catchment size improves, R^2 increasing from 0.6914 to 0.9066 in Fig C(c) versus Fig D(c). In contrast to the original analysis in Fig C, accounting for a potential 5-fold reduction in LFD test rates weakens the correlation, with R^2 decreasing from 0.9066 to 0.7472 in Fig D(c) versus Fig D(d).

Overall, we observe that the wastewater SARS-CoV-2 detection frequency observed at the five different near-source sites did not correlate with case numbers alone. Correlation between detection frequency and sewer catchment size was observed, improved when multiplying catchment size by case numbers (as an indicator of likely viral prevalence). The linear regressions in Fig C(c),(d) and Fig D(c),(d) reach the x-axis (zero frequency of detection) at approximately log(cases·catchment size) = -2, representing a 1% probability of a new case within the catchment each day. The linear regressions reach 50% detection frequency at approximately log(cases·catchment size) = -0.4, representing a 40% probability of a new case within the catchment each day. The approximately linear relationship is not anticipated to hold when wastewater detection frequencies approach 0% and 100% respectively. We would expect that at low viral prevalence and small catchments, where it is unlikely that we will find multiple COVID-19 carriers with overlapping shedding profiles, there will be a linear relationship between the frequency of wastewater detection and case numbers, catchment size, or the product of the two. Where case numbers or catchment size become sufficiently large that the shedding profiles from many individuals overlap, increases in the frequency of detection with log(case numbers·catchment size) will slow down or plateau. And as the frequency of detection approaches 100%, further increases in case numbers or catchment size will cease to have much influence at all on the frequency of detection. We consequently expect a sigmoidal profile between log(case numbers·catchment size) and the frequency of detection.

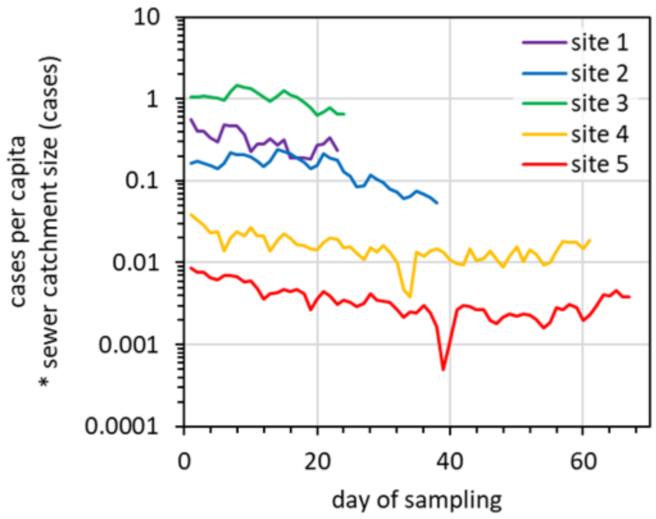

Fig E: Theoretical number of new cases within the catchment population each day of sampling, obtained by multiplying the case number rate by the sewer catchment size.

Whilst the sites tested during 2022 all showed the strongest and more frequent wastewater SARS-CoV-2 detections when national surveillance COVID-19 case numbers were greatest, the sites tested during 2023 (sites 4 and 5) did not. This may be due to a decoupling of large-scale trends from near-source observations during periods of low viral prevalence. By multiplying COVID-19 case numbers by the catchment size, we can indicate the relative likelihood of finding a significant number of SARS-CoV-2 shedders within the catchment population (Fig E). The correlation of wastewater detections and case numbers is greatest for site 3 (the museum, main text Figure 8) which showed the greatest number of cases per sewershed (estimated by the multiplication of case numbers by catchment population). This was the only site where we can estimate at least one new COVID-19 carrier within the population each day, and where we would be consequently be statistically confident of finding a small population of shedders (site 3, Fig E). In contrast, based on case numbers and catchment size, on any given day we would expect a <1% chance of an individual within the site 4 catchment (university) and <0.1% chance of an individual within the site 5 (care home) catchment acquiring COVID-19. Ergo on any given week we are unlikely to have new shedders within the catchment. The care home is the only site where wastewater SARS-CoV-2 detects were not more likely on days where COVID-19 case numbers were higher. The greater difference between near-source wastewater results from national surveillance at this site is likely due to both the small catchment size, but also the nature of the site. The care home represents a semi-closed site, with permanent residents not leaving the care home grounds, and staff taking extra precautions to minimise the transmission of infectious diseases to the vulnerable population. Consequently, we would expect the greatest deviation between near-source wastewater data and national surveillance results at this site.

12. Distribution of Positive Detections Between Pathogens Monitored at the University and Care Home

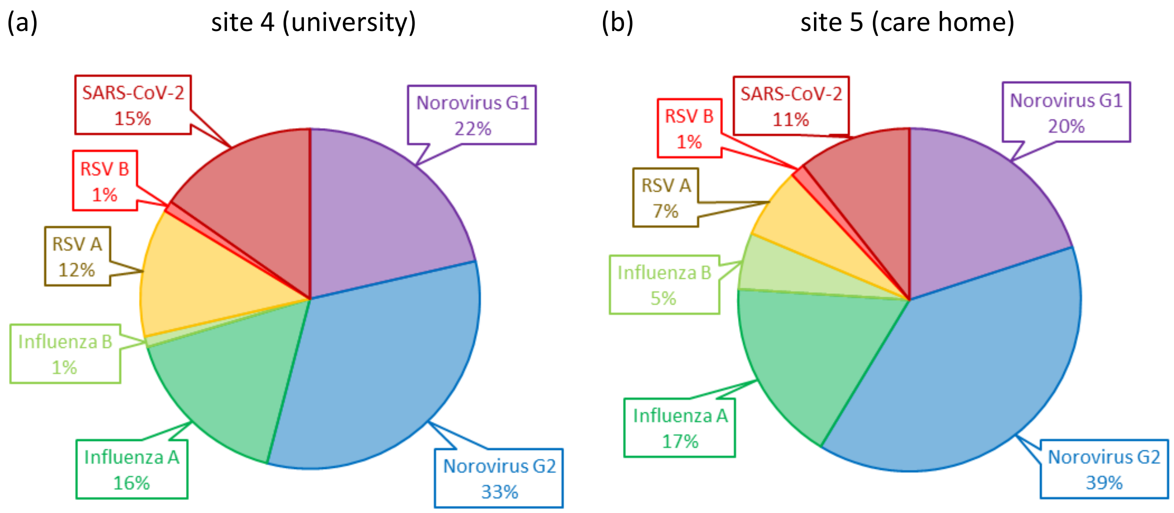

Fig F: Distribution of wastewater detections between the different pathogens monitored during 2023 at (a) site 4 (university), and (b) site 5 (care home). Percentages indicate the number of detections for each pathogen target as a proportion of all detections.

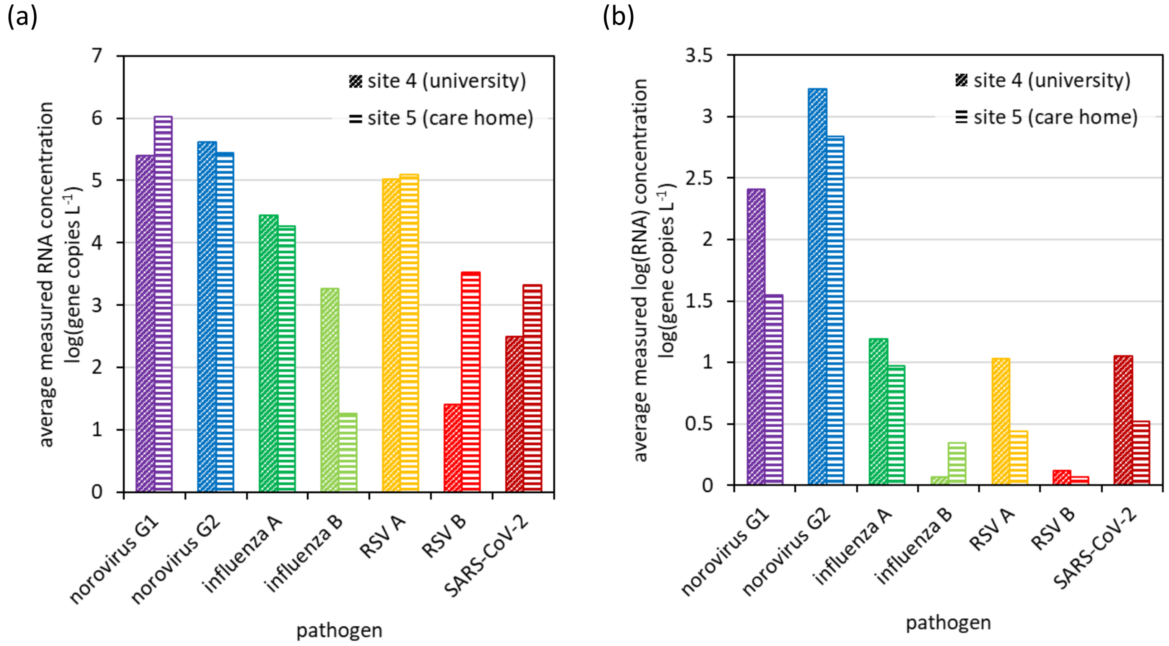

Fig G: (a) Average measured RNA concentration and (b) average measured log(RNA) concentration, for the different pathogens monitored during 2023 at site 4 (university, cross-hatched bars) and site 5 (care home, bars with horizontal stripes).

13. Box Plots for Norovirus, Influenza A Virus, and RSV A

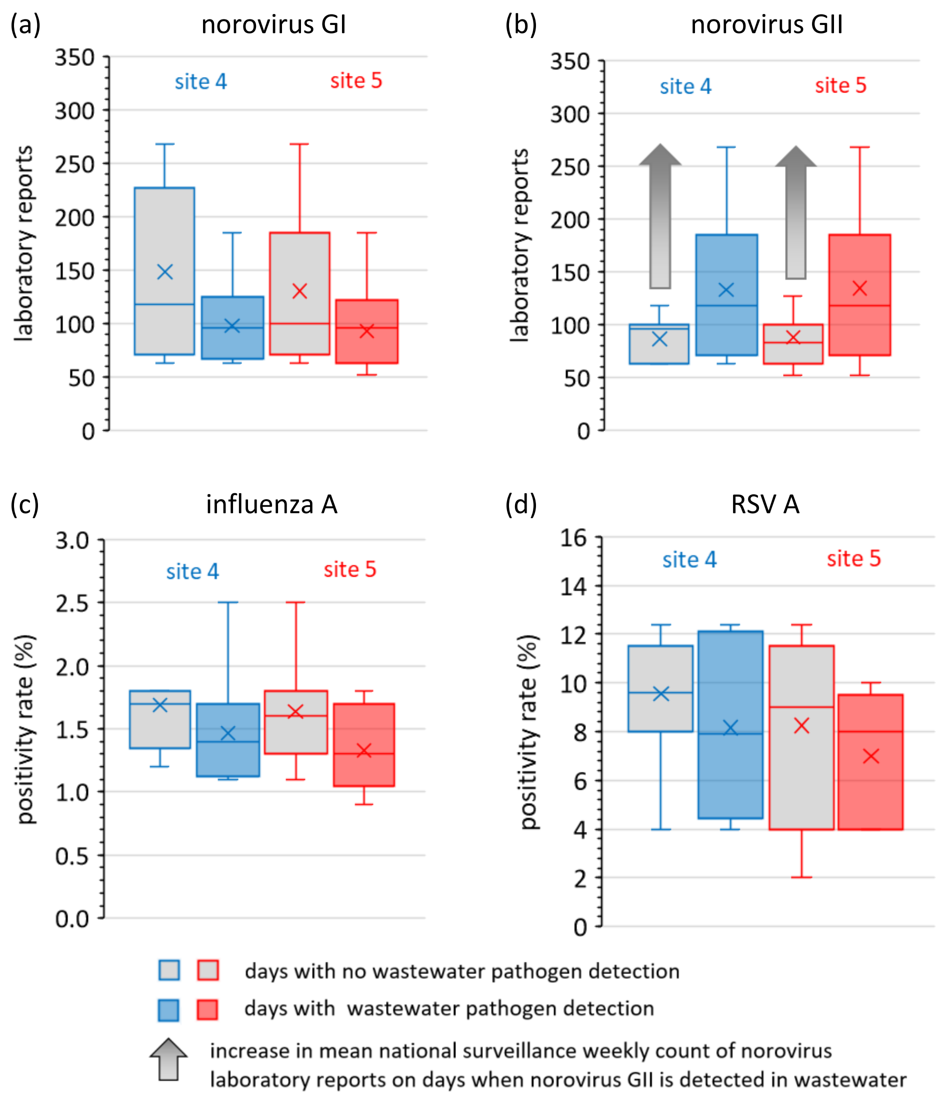

Fig H: Box plots to show the difference between (a) norovirus GI, (b) norovirus GII, (c) influenza A, and (d) RSV A viral prevalence as indicated by the UK national surveillance programme's published data, on days with and without wastewater detection of the same pathogen. Data is presented for days without (grey shading) and with (colour shading) wastewater viral detection, and the 2023 sites are the university (blue) and the care home (red). The box plots indicate the minimum, first quartile, median, average, third quartile, and maximum values of each data set.

14. Daily Changes in SARS-CoV-2

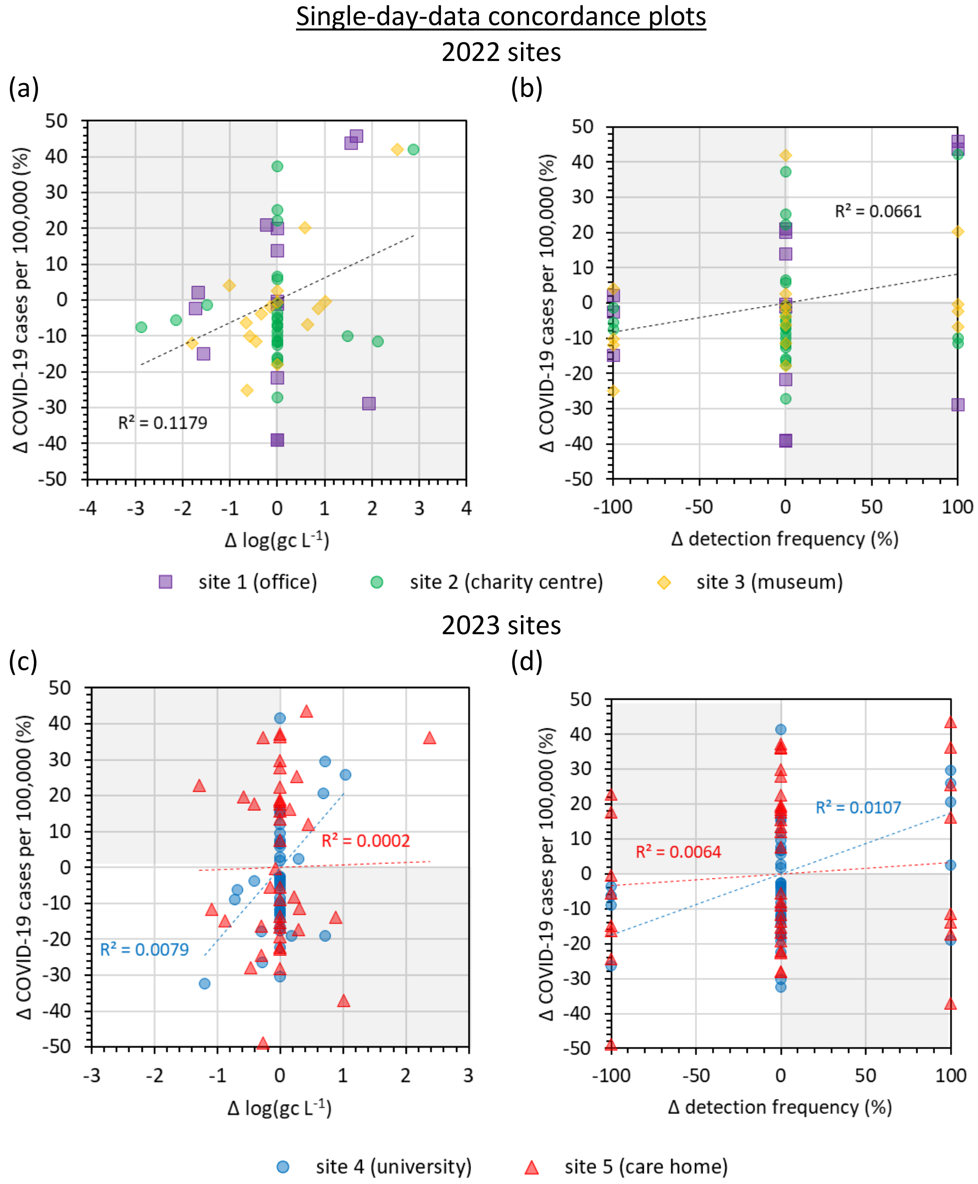

Fig I: Plots showing the concordance between the day-to-day change in wastewater SARS-CoV-2 measurements (RNA concentration or presence/absence) and the day-to-day change in COVID-19 case numbers. Each data point is the measured value of the parameter at day X, subtract the measured value of that same parameter at day (X-1).

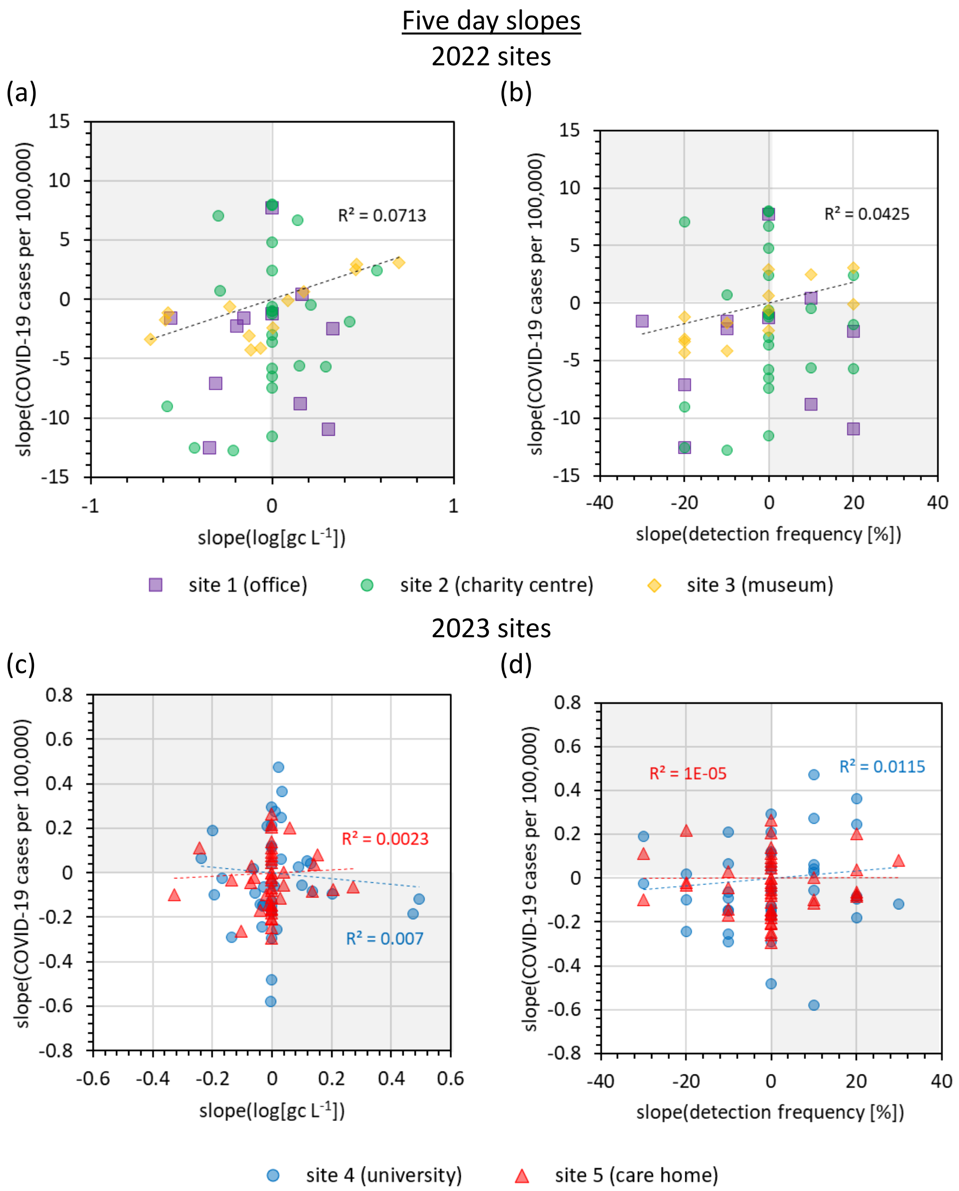

Fig J: Plots showing the concordance between the five-day slope of wastewater SARS-CoV-2 measurements (RNA concentration or presence/absence) and the five-day slope COVID-19 case numbers. Slopes were calculated as per a simple linear regression. Each data point is the value of the slope in the measured parameters, using five data points between day X and day (X-4).

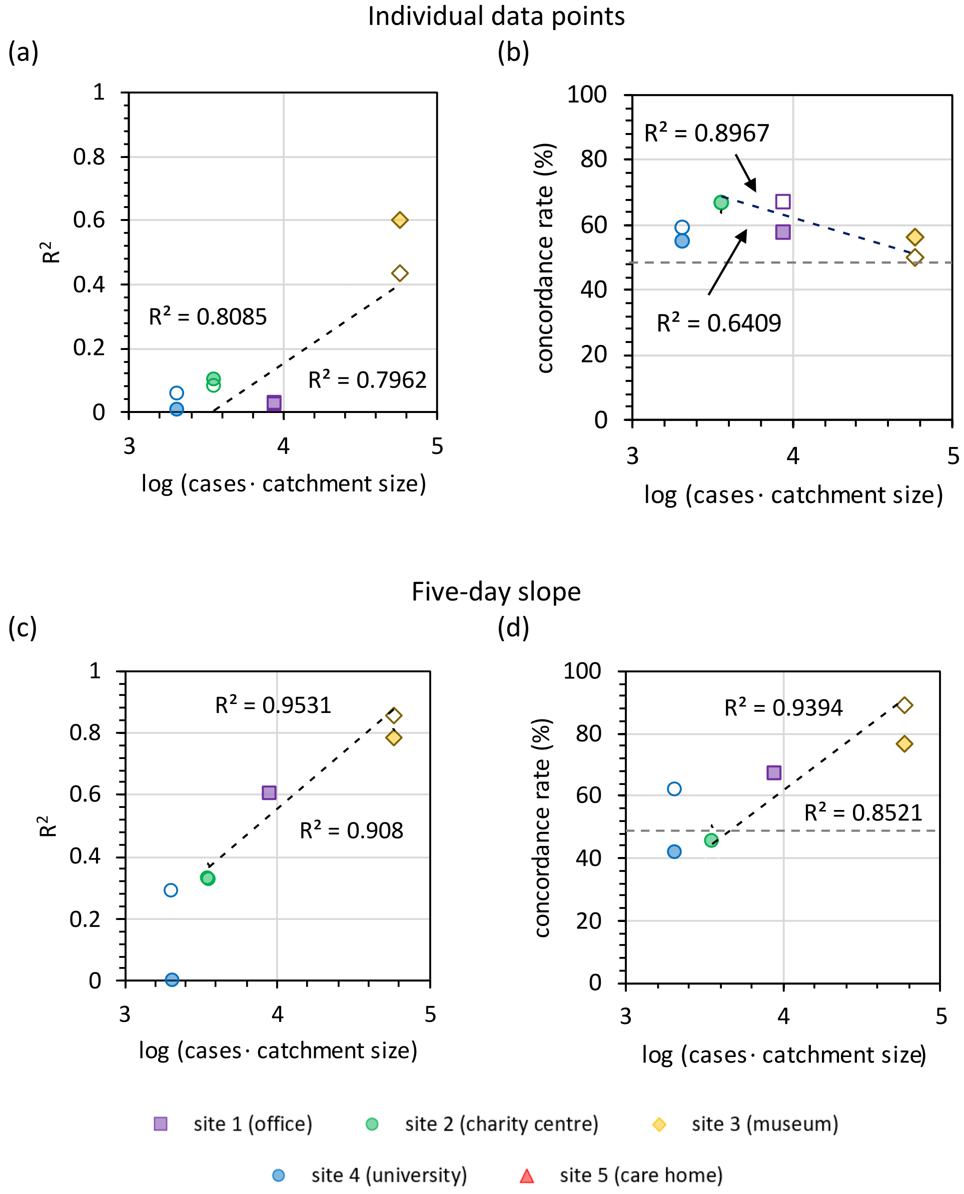

Fig K: Correlation and correspondence between near-source wastewater SARS-CoV-2 data and national surveillance COVID-19 data, using individual day data points in isolation, and using a five-day linear regression. Day-to-day (a) correlation and (b) concordance between the individual measurement of wastewater RNA concentration (solid shapes) and detection frequency (empty shapes) versus COVID-19 case numbers. Each day's value was considered using a single measurement in isolation (i.e. one concentration measurement, or 100% versus 0% detection frequency for a positive or a negative detection). (c) Correlation and (d) concordance for the five-day slope (linear regression) of wastewater RNA concentration (solid shapes) and detection frequency (empty shapes) versus COVID-19 case numbers. Each day's value was calculated using a five-point linear regression (i.e. that day's measurement, and the preceding four days' worth of data). Data is presented for site 1 (office, purple squares), site 2 (charity centre, green circles), site 3 (museum, yellow diamonds) and site 4 (university, blue circles). Site 4, sampled in 2023, would likely also fit the linear regression between concordance rates and log(cases·catchment size) if the testing bias between 2022 and 2023 were accounted for, inflating the case numbers by a factor of five. Data for site 5 (care home) is not presented: negative correlations were observed and there was no concordance.

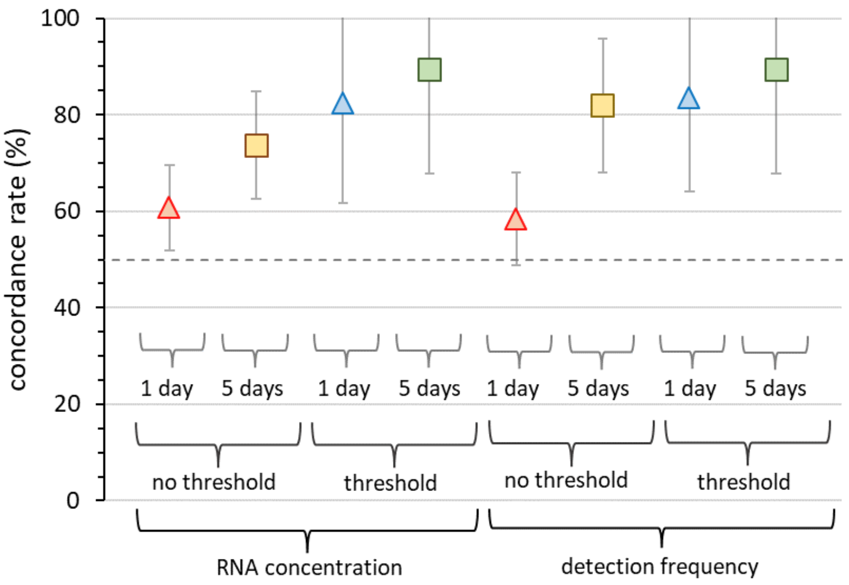

Fig L: Concordance between day-to-day movement in wastewater SARS-CoV-2 surveillance and COVID-19 case numbers. Concordance rates were calculated considering two consecutive daily data points ('1 day'), and considering a day-to-day change in the five-day moving average of RNA concentration, RNA detection frequency, and case numbers. A 10% threshold was applied, removing any data points where SARS-CoV-2 RNA concentration, detection frequency, or COVID-19 case numbers varied by less than 10%. Markers indicate the average concordance rate calculated between sites 1, 2, 3 and 4, and error bars indicate the standard deviation within this average. Site 5 is omitted from the analysis, given that a negative correlation was observed between wastewater SARS-CoV-2 detection and COVID-19 case numbers.

15. Week-by-Week Correlation and Concordance in SARS-CoV-2

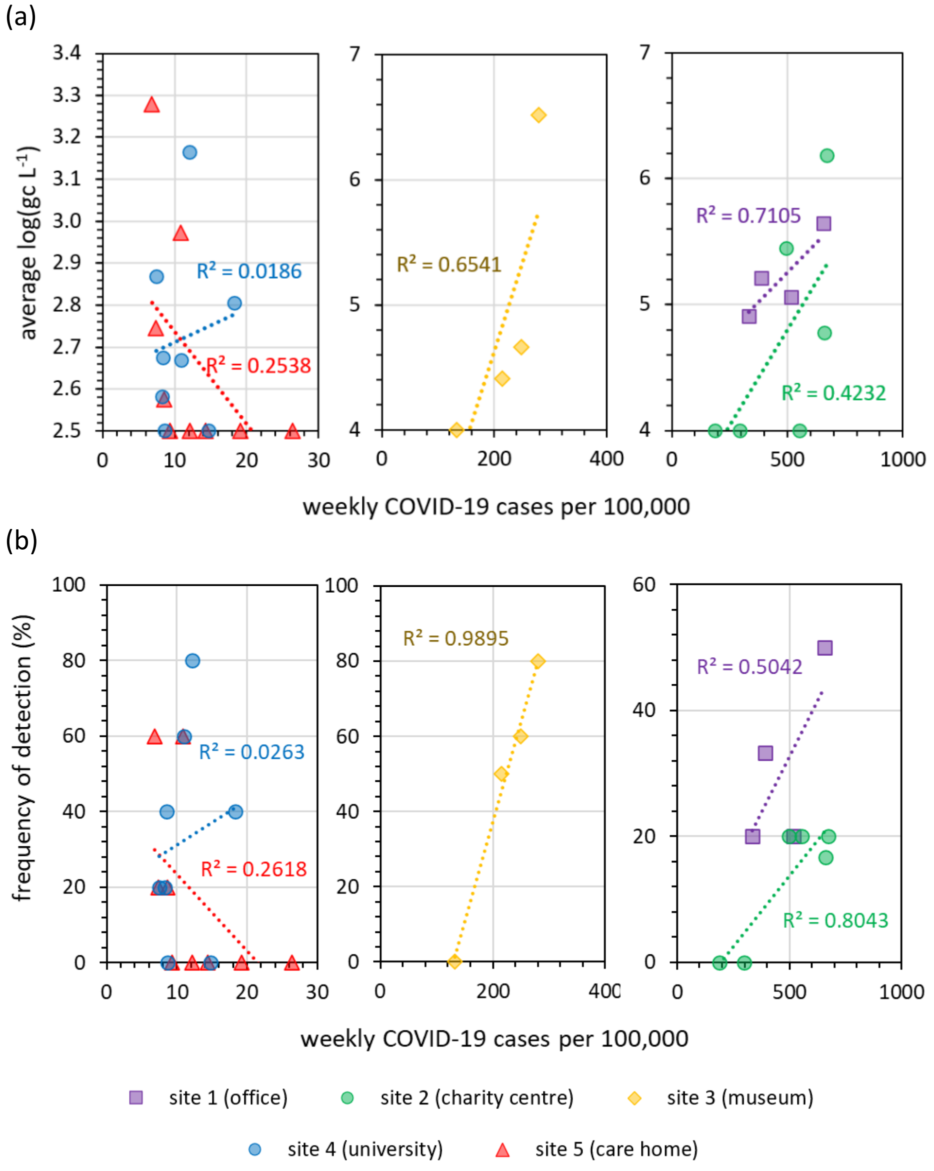

Fig M: Correlation between the weekly number of COVID-19 cases (per 100,000 people) and (a) the weekly average log(SARS-CoV-2 RNA concentration [gene copies L^{-1}]) or (b) the weekly SARS-CoV-2 detection frequency.

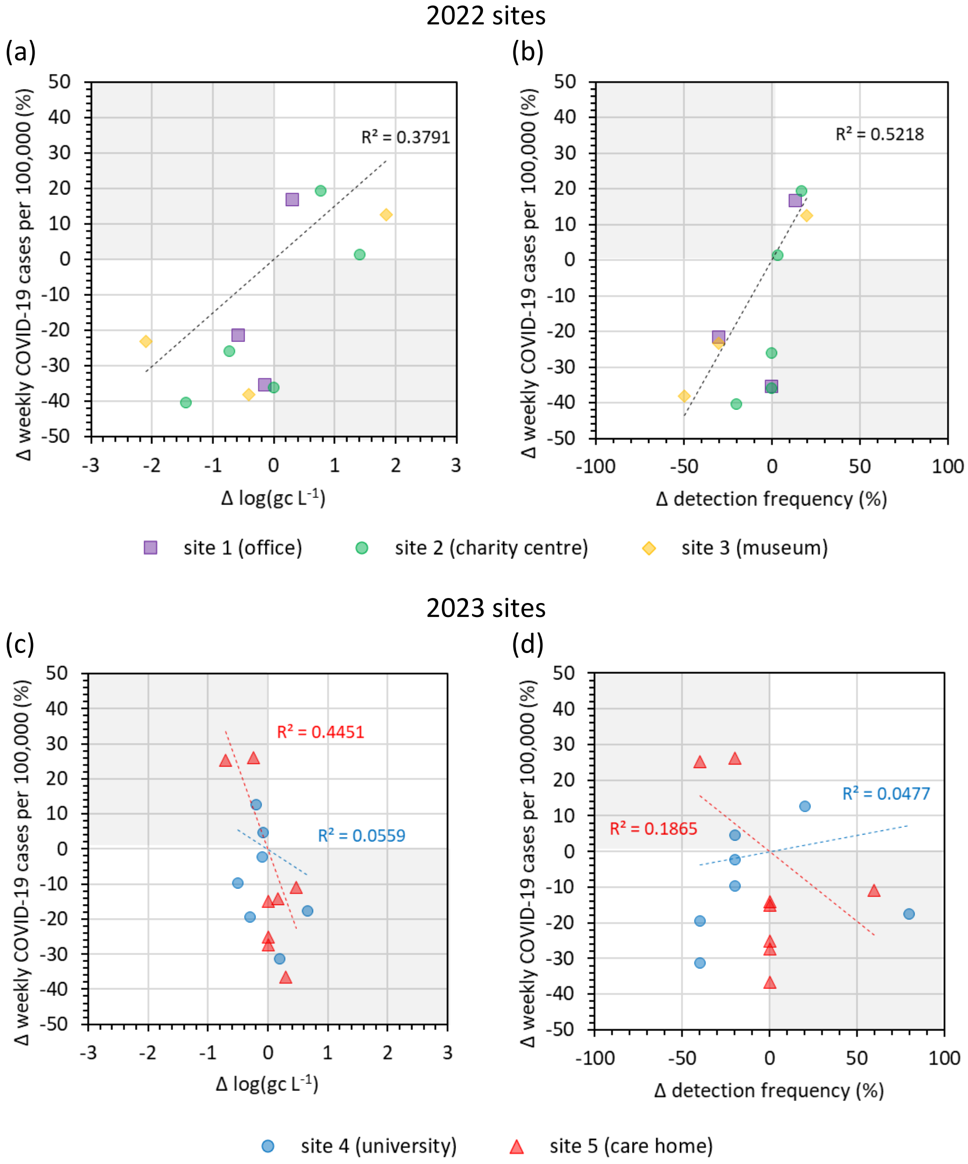

Fig N: Concordance plots for the weekly change in near-source wastewater SARS-CoV-2 RNA concentrations (left-hand panels) and detection frequency (right-hand panels) with COVID-19 case numbers (national surveillance).

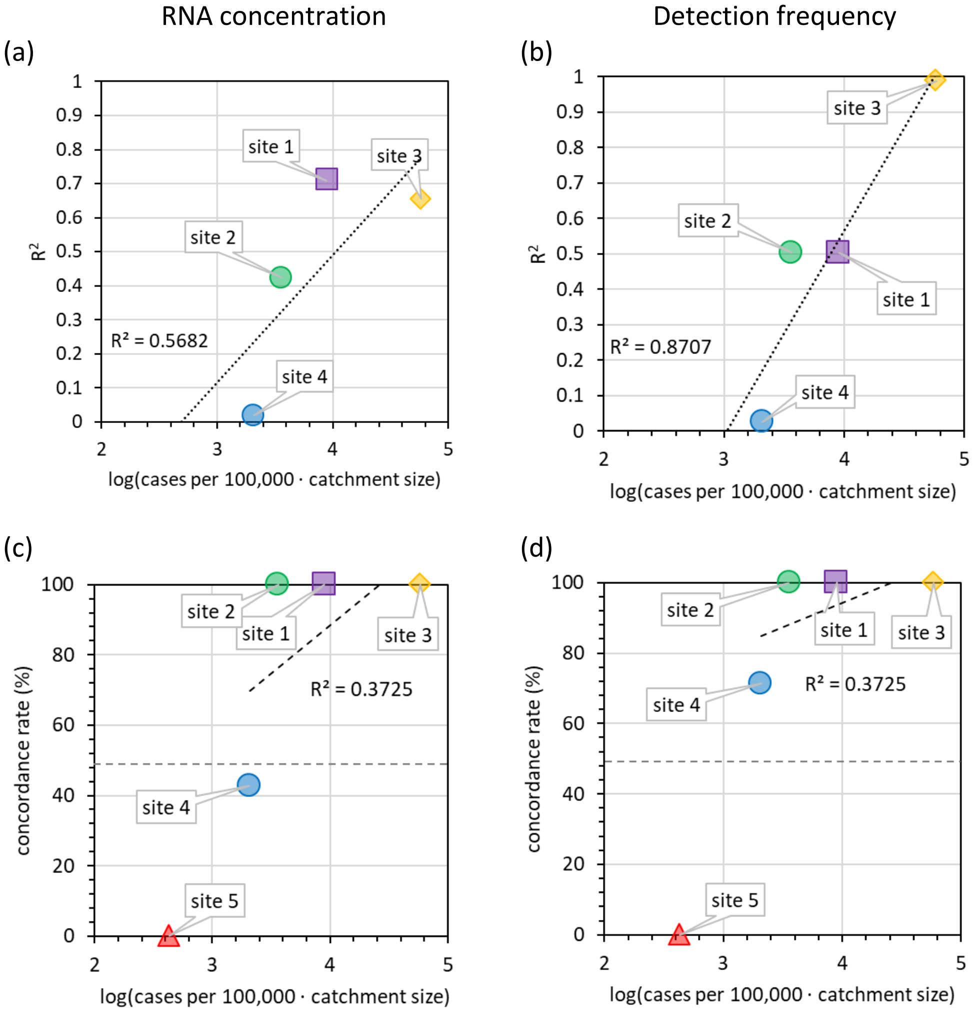

Fig O: Week-by-week data analysis and the influence of catchment size and viral prevalence on the correlation and concordance between wastewater SARS-CoV-2 analysis and COVID-19 case numbers. Site 5 (care home) is excluded from figures (a) and (b) since a negative correlation was observed. The x-axis indicates the logarithm of the multiplication product of national surveillance COVID-19 case numbers (per 100,000 people per day) and the sewershed catchment size (population). In this figure, concordance rates were calculated exclusive of weeks with consecutive wastewater non-detects, whilst the main text presents concordance rates inclusive of weeks with consecutive wastewater non-detects. Consecutive non-detects would increase the number of total pairs, despite no increase in the number of discordant pairs. Consequently concordance rates for sites 1 and 2 are greater in panel (d) of this figure versus the main text.

17. Week-by-Week Correlation and Concordance in Norovirus GII

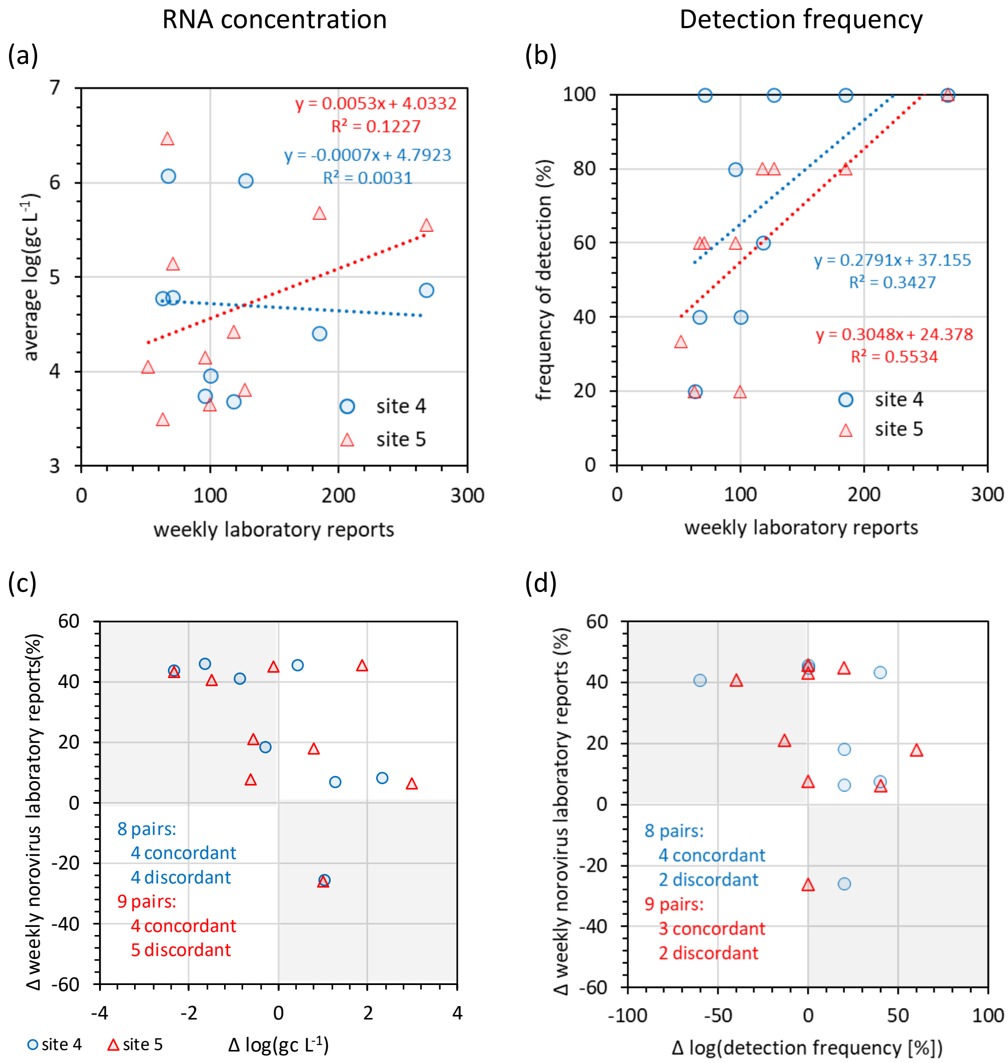

Fig P: (a,b) Correlation and (c,d) concordance plots (or lack of concordance) for wastewater norovirus GII detection versus national surveillance norovirus laboratory report numbers. Wastewater data was used as the weekly average measured RNA concentrations (left-hand panels) and weekly detection frequency (right-hand panels)

18. Comparison of Correlation and Concordance Metrics

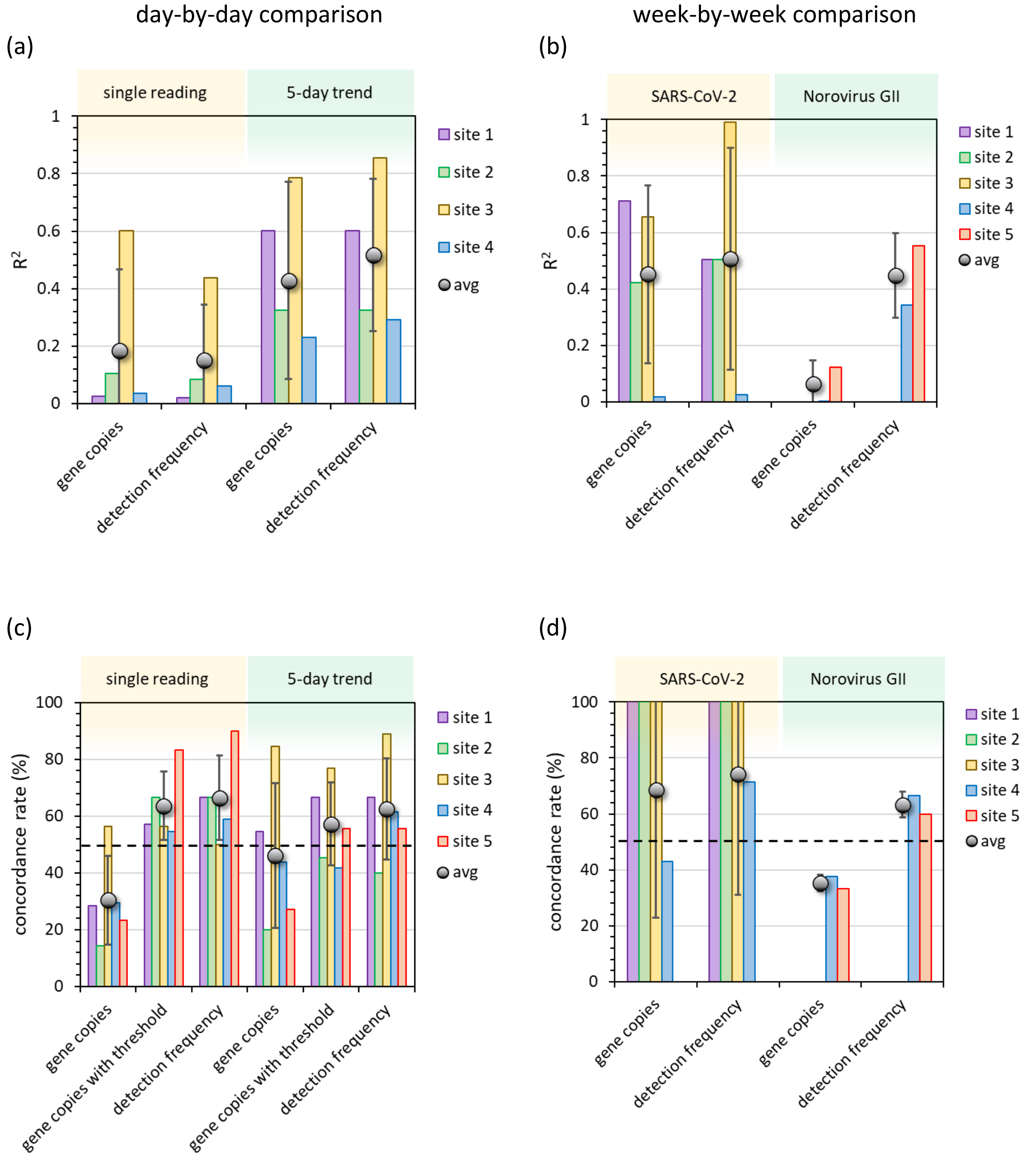

Fig Q: Final comparison of correlation coefficients and concordance rates between near-source wastewater data and national surveillance reports (both SARS-CoV-2 and norovirus GII). "Single reading" refers to day-to-day SARS-CoV-2 measurements, where the change in the measured parameter is calculated using the value at day X, minus the value at day (X-1). "5-day trend" refers to a one week trend in SARS-CoV-2 measurements, calculated using the slope of the linear regression fit to five consecutive daily measurements, from day X to day (X-4). "Week-by-week" comparison refers to weekly aggregates (e.g. the five-day average of wastewater measurements taken between Monday and Friday each week, and the weekly total number of COVID-19 cases). R^2 values are not shown for SARS-CoV-2 detection at site 5 (care home) as the linear regression gave a negative correlation. Sites 1-3 are not included within the norovirus GII plots, as norovirus was only measured at sites 4 and 5. Other pathogens are not presented, since no concordance was observed using box plot analysis (Fig H).

The correlation between the day-to-day change in national surveillance case numbers and wastewater measurements (RNA concentration and presence/absence) is poor when considering data points in isolation (Fig Q(a), "single reading"). A correlation can be obtained when we instead consider the slope (linear regression) of the last five data points (Fig Q(a), "5-day trend"). This appears to be due to the five day slope smoothing out the data set and revealing the biasing of both wastewater detects and case numbers towards the start of each working week. The fact that correlation isn't picked up when considering the change between just two daily data points is perhaps due to the weekday effect being weaker in the case number data than the wastewater data (potentially due to a variable lag time between reported case numbers versus viral load, as presented in Fig B). The correlation of SARS-CoV-2 wastewater data with national surveillance significantly improves for some sites, but becomes very poor for site 4 (university) when considering week-to-week changes, Fig Q(b). Overall, the average correlation coefficients (R^2) do not significantly change (though the variance increases). By considering week-to-week changes in place of day-to-day changes, we remove the contribution of Monday biasing towards the positive correlation, and the correlation observed is therefore instead due to correlation between wastewater detections and case numbers over longer timescales (i.e. changes in the weeks-to-months timescale). The poor correlation at site 4 is attributed to the low SARS-CoV-2 prevalence at the time of sampling. Correlation coefficients for norovirus GII at sites 4 and 5 are weak, presumably due to low norovirus GII prevalence (discussed in the main text). The data sets show very poor concordance between wastewater data and national surveillance data when considering the change between two consecutive daily data points only (Fig Q(c), "single reading"). The concordance is improved when using a threshold to remove data points where parameters change by less than 10% (reducing the number of pairs that are neither concordant nor discordant). For the 2022 sites, concordance is significantly improved when comparing the week-on-week change instead of day-on-day with 100% concordance for sites 1 and 3, Fig Q(d). This is likely due to the significant swing in case numbers and wastewater detections on a weeks-to-months timescale during COVID-19 variant waves. Concordance was very poor for norovirus GII at sites 4 and 5, presumably due to low prevalence and the high variability in wastewater detections, perhaps due to super shedder effects, Fig Q(d). Overall, the observation that correlation improves both when replacing a day-to-day change with a five-day linear regression, and when replacing day-to-day changes with week-to-week changes, indicates that correlations between near-source wastewater and national surveillance data sets are due to both short-term weekday periodicity effects as well as longer term macroscale variations in viral prevalence.

19. Chemical Characterisation (pH and NH_3) at Sites 4 and 5

Nitrogen concentrations (as NH_3-N) were recorded using a Hanna Instruments HI-700 ammonia low range colorimeter checker. Where necessary, samples were diluted with deionised water to give a reading within the 0 to 3 mg L^{-1} detection window. Measurements were calibrated using a two-point calibration curve, with standards at 0 and 1.5 mg L^{-1}. pH readings were recorded using a pH meter, with measurements calibrated using a three-point calibration curve (at pH 4, 7 and 10).

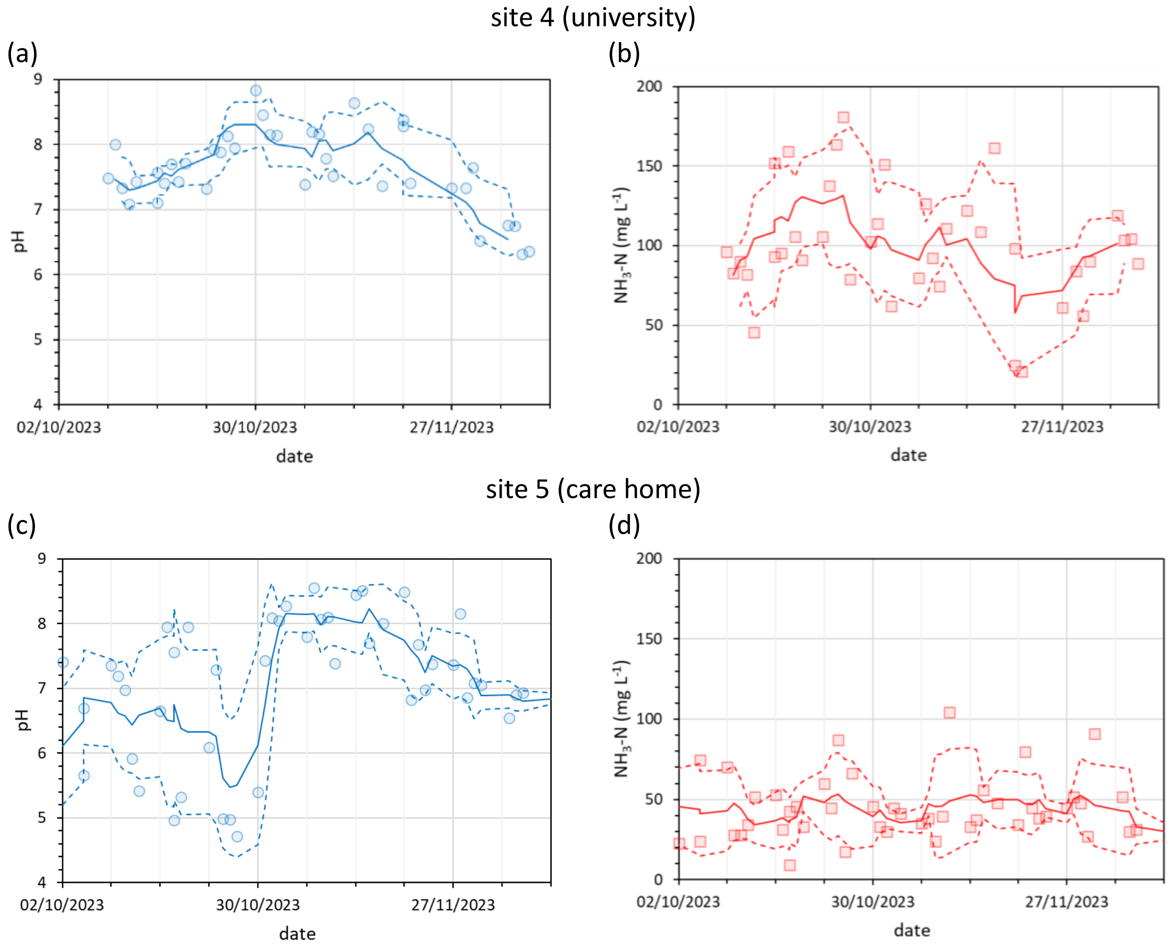

Fig R: Partial chemical characterisation (pH and NH_3) at the university and care home. Solid lines indicate the five-point moving average, whilst dashed lines indicate the average plus and minus one standard deviation. An outlier sample from site 5 has been omitted from the time series, where a low pH reading of 5 continuously decreased towards zero during analysis with the pH meter, and a high NH_3-N reading of 272 mg L^{-1} was recorded, greater than any other sample.

The time series of pH and nitrogen (as ammonia) recorded in the wastewater effluent from site 4 (university) and site 5 (care home) are presented in Fig R. The average pH recorded at the university was 7.6 ± 0.6, greater than the average pH of 6.8 ± 1.2 at the care home. The average NH_3-N concentration recorded at the university was 100 ± 36 mg L^{-1}, again greater than the average of 43 ± 34 mg L^{-1} at the care home. At the university, a neutral-alkaline pH became slightly acidic during the final two weeks of the field study. Ammonia concentrations showed no significant trend. At the care home, a slightly acidic average pH during the first month of field work, with pH 5-6 measurements for half the samples, increased to pH 8 in the second month of field work and then drifted to 7. Ammonia concentrations showed no similar trend. A septic tank upstream of the sampling point

temporarily held sewage, and septic sewage is often more acidic than fresh sewage. The acidic pH measurements during the first month of field work might be linked to the discharged of aged septic sewage. In both the overall time series, and week-by-week data analysis, the variation in pH and relative variation in ammonia readings were greater at the care home than the university. This is likely due to the smaller catchment size at the care home, with changes in the day-by-day and week-to-week activities on-site having a greater impact on the composition of the wastewater effluent. No correlation was observed between pH and NH_3-N measurements at either site (Fig S).

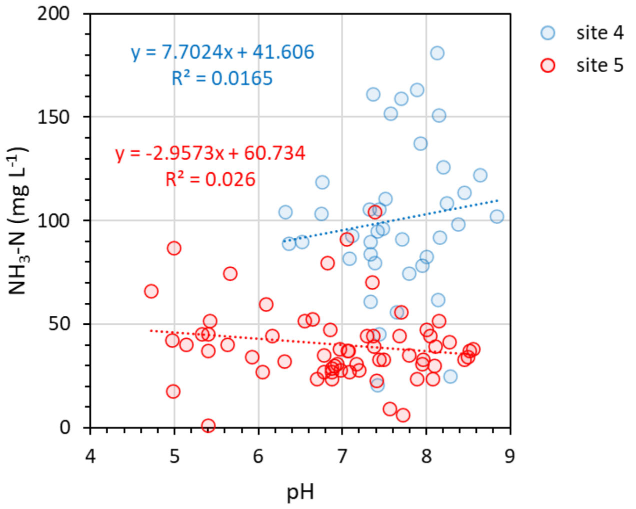

Fig S: Lack of a correlation between NH_3-N and pH at site 4 (university) and site 5 (care home).

20. Data Communication to End Users

At sites 1 and 2, results were communicated via short daily email updates. At site 3, daily emails were sent to the museum's facilities management team, which linked to a spreadsheet containing the full timeline of wastewater results. For sites 4 and 5, an online data dashboard was provided to the data users (a consortium of site operations and wellbeing professionals at the university, and the managers and directors at the care home). The dashboard presented a calendar view to show detection results for each pathogen target, with the data collected in 3 buckets to represent 'low', 'medium' and 'high' viral prevalence. The low prevalence bucket included both non-detects and detections with C_t values of 35 and higher, the medium detection bucket included detections with C_t values from 30 to 35, and the high detection bucket included detections with C_t values below 30. An estimated 'risk level' was then presented based upon an exponential moving average of the latest data. Towards the end of the field work, data users also received automated SMS alerts (text messages) to indicate when a medium or strong detection had occurred.

21. Feedback from Stakeholders and Data Users

Table 4: Summary of data users and the operational implementation of wastewater data. The office provided data indicating the days with positive COVID-19 lateral flow test results. The charity centre did not record COVID-19 related sickness, instead employing a handheld non-contact infrared thermometer to ensure individuals admitted to site were not experiencing elevated temperatures. The museum did not provide COVID-19 related sickness data. The university indicated the weeks where different symptoms had been observed from the site's staff, however data relating to the broader community (e.g. students and researchers) was not available. The care home did not encounter obvious signs of respiratory of gastro-intestinal outbreaks during the study period.

Use	Data user	Pre-existing local monitoring methods	Wastewater data correlated to local observations?	Implementation of wastewater data
Office	Office manager	Daily lateral flow tests	Yes	•Identifying risk when carriers escaped diagnostic screening tests
Charity Centre	Administrative office manager	Daily non-contact infrared thermometer readings	N/A	•Negative results gave confidence that on-site hygiene, sanitation, and occupancy limits are keeping risk low
Museum	Facilities and operations manager	Self-reported COVID-19 cases	N/A	•(Data not actioned due to lack of sufficient baselining period prior to observed wastewater peak)
University	•Operations manager<br>•Healthy, Safety and Wellbeing lead<br>•Communications Manager	Self-reported staff absences	Yes	•Increased cleaning<br>•Information campaign across campus<br>•Hygiene information posters in all bathrooms
Care home	Director	Observation of residents	No	•(Data not actioned due to lack of obvious norovirus-like cases)

The availability of diagnostic or symptom-based case data varied between the different sites, and varied across the different stages of the COVID-19 pandemic. At site 1 (office), field work was conducted shortly after the Omicron BA.1 peak [54] and staff were required to take a lateral flow device (LFD) test each day. At site 2 (charity centre), field work was conducted during the Omicron BA.2 peak [54] and instead of a LFD test, visitors were only required to give a temperature reading. For the three other field work studies occurring after, no screening tools were used to manage site access. The site-specific data available to site managers was limited to self-reported symptoms and sick days taken (as well as door counts or headcounts at the museum and university).

End-user engagement varied between sites, with more data exploration and implementation at the two larger sites (the university and the museum). Potentially, this is due to each site having different data users, with different roles, responsibilities, and professional interests. A summary is provided in Table 4. At sites 1, 2 and 4, data users were not responsible for health and wellbeing; job responsibilities were very hands on and reactionary. The opportunity to fully engage during a research project with quantitative wastewater data appears limited for individuals in these types of roles, and data users would likely benefit from clearly established guideline limits. In spite of this, at the office, the data user was able to connect positive wastewater SARS-CoV-2 detects to the presence of external site visitors, who unlike permanent employees were not mandated to take daily LFD tests, and identify potential shortcomings in the company's testing policy.

Greater engagement was observed at the museum and university, where the data users held more strategic roles, offering more time to think critically about the data presented. Interviews with the data user at the museum indicated the need to establish robust thresholds to convert near-source wastewater data into low and high risk levels. The main limitation to actioning the wastewater data arose due to the observed SARS-CoV-2 peak occurring in the second week of a four week field study: There was little historical baselining data during a periodic of comparatively lower COVID-19 prevalence to contextualise the BA.4/BA.5 peak. The stakeholder interview established that near-source wastewater data can be made more actionable by running longer pilots with more time to establish nucleic acid concentration thresholds that correspond to low and high pathogen prevalence. Alternatively, the data user would find near-source wastewater data easier to action if guideline limits were provided, as they were familiar with government-established guideline limits for Legionella, which is routinely tested for at the museum. The data user thought that actionability might be increased by establishing a control hierarchy, with changing risk levels mapped onto a matrix, escalating as far as a total shutdown of the site.

Data users at the university comprised a small consortium of stakeholders, which included strategic roles as well as operational roles (Table 4). We again encountered the topic of baselining wastewater data before taking action. During this longer field study we were able to collect 5 weeks of baselining data, after which data users decided to act upon a rising norovirus signal. The team scheduled more intensive cleaning (especially for touchpoints, e.g. door handles and bannisters) and implemented an information campaign in all toilets (posters warning of winter sickness, and recommending thorough handwashing). Our observation that a certain baselining period is necessary to develop data user's confidence in taking actions on the basis of near-source wastewater data is similar to observations made during municipal-scale surveillance programmes setup to combat the COVID-19 pandemic, where it took time for government decision makers to understand how municipal wastewater surveillance data might best inform decision making [55].

At the museum, motivation to engage with wastewater data was linked to a desire to understand local viral risk, rather than relying on national-scale surveillance. The data user expressed an interest to sample different locations within the overall campus, to provide granular detail and separate risk

levels according to building function. Besides self-reported COVID-19 sickness, the data user had relied on national surveillance including the Zoe Health Study (a UK smartphone app-based study that tracked COVID-19 prevalence by asking users to indicate their location, symptoms linked to COVID-19, and any test results).

Data users at the university emphasised how they found the wastewater pilot to be non-invasive, with no impact on daily operations. Other comments included a surprise that despite advertising the wastewater monitoring pilot on campus, no students or members of staff expressed concern.

22. References

1. Colman E, Puspitarani GA, Enright J, Kao RR. Ascertainment rate of SARS-CoV-2 infections from healthcare and community testing in the UK. J Theor Biol. 2023;558. doi:10.1016/j.jtbi.2022.111333

2. Understanding reported COVID-19 cases in England following changes to testing, between November 2021 and April 2022. doi:10.1101/2022.06.28.22276549

3. England Summary | Coronavirus (COVID-19) in the UK. [cited 1 Feb 2024]. Available: https://coronavirus.data.gov.uk/

4. Coronavirus (COVID-19) Weekly Update - London Datastore. [cited 1 Feb 2024]. Available: https://data.london.gov.uk/dataset/coronavirus--covid-19--cases

5. Jones DL, Baluja MQ, Graham DW, Corbishley A, McDonald JE, Malham SK, et al. Shedding of SARS-CoV-2 in feces and urine and its potential role in person-to-person transmission and the environment-based spread of COVID-19. Science of the Total Environment. 2020;749. doi:10.1016/j.scitotenv.2020.141364

6. Schmitz BW, Innes GK, Prasek SM, Betancourt WQ, Stark ER, Foster AR, et al. Enumerating asymptomatic COVID-19 cases and estimating SARS-CoV-2 fecal shedding rates via wastewater-based epidemiology. Science of the Total Environment. 2021;801. doi:10.1016/j.scitotenv.2021.149794

7. Xin H, Wang Z, Feng S, Sun Z, Yu L, Cowling BJ, et al. Transmission dynamics of SARS-CoV-2 Omicron variant infections in Hangzhou, Zhejiang, China, January-February 2022. International Journal of Infectious Diseases. 2023;126: 132–135. doi:10.1016/j.ijid.2022.10.033

8. Puhach O, Meyer B, Eckerle I. SARS-CoV-2 viral load and shedding kinetics. Nature Reviews Microbiology. Nature Research; 2023. pp. 147–161. doi:10.1038/s41579-022-00822-w

9. Ward T, Glaser A, Overton CE, Carpenter B, Gent N, Seale AC. Replacement dynamics and the pathogenesis of the Alpha, Delta and Omicron variants of SARS-CoV-2. Epidemiol Infect. 2023;151. doi:10.1017/S0950268822001935

10. Tanaka H, Ogata T, Shibata T, Nagai H, Takahashi Y, Kinoshita M, et al. Shorter Incubation Period among COVID-19 Cases with the BA.1 Omicron Variant. Int J Environ Res Public Health. 2022;19. doi:10.3390/ijerph19106330

11. Park SW, Sun K, Abbott S, Sender R, Bar-On Y, Weitz JS, et al. Inferring the differences in incubation-period and generation-interval distributions of the Delta and Omicron variants of SARS-CoV-2. doi:10.1101/2022.07.02.22277186

12. Xu X, Wu Y, Kummer AG, Zhao Y, Hu Z, Wang Y, et al. Assessing changes in incubation period, serial interval, and generation time of SARS-CoV-2 variants of concern: a systematic review and meta-analysis. BMC Med. 2023;21. doi:10.1186/s12916-023-03070-8

13. Ogata T, Tanaka H. SARS-CoV-2 Incubation Period during the Omicron BA.5- Dominant Period in Japan. Emerg Infect Dis. 2023;29: 595–598. doi:10.3201/eid2903.221360

14. Li X, Tam AR, Chu WM, Chan WM, Ip JD, Chu AWH, et al. Risk Factors for Slow Viral Decline in COVID-19 Patients during the 2022 Omicron Wave. Viruses. 2022;14. doi:10.3390/v14081714

15. Hay JA, Kissler SM, Fauver JR, Mack C, Tai CG, Samant RM, et al. Quantifying the impact of immune history and variant on SARS-CoV-2 viral kinetics and infection rebound: A retrospective cohort study. Elife. 2022;11. doi:10.7554/eLife.81849

16. de Michelena P, Olea B, Torres I, González-Candelas F, Navarro D. SARS-CoV-2 RNA load in nasopharyngeal specimens from outpatients with breakthrough COVID-19 due to Omicron BA.1 and BA.2. J Med Virol. 2022;94: 5836–5840. doi:10.1002/jmv.28079

17. Kandel C, Lee Y, Taylor M, Llanes A, McCready J, Crowl G, et al. Viral dynamics of the SARS-CoV-2 Omicron Variant among household contacts with 2 or 3 COVID-19 vaccine doses. Journal of Infection. 2022;85: 666–670. doi:10.1016/j.jinf.2022.10.027

18. Funk S, Abbott S. Cycle threshold values in symptomatic COVID-19 cases in England. 2022. doi:10.1101/2022.06.13.22276321

19. UK Health Security Agency. Evaluation of lateral flow device performance within the National Testing Programme. 2022.

20. Mellou K, Sapounas S, Panagoulias I, Gkova M, Papadima K, Andreopoulou A, et al. Time Lag between COVID-19 Diagnosis and Symptoms Onset for Different Population Groups: Evidence That Self-Testing in Schools Was Associated with Timely Diagnosis among Children. Life. 2022;12. doi:10.3390/life12091305

21. Núñez I, Belaunzarán-Zamudio PF, Caro-Vega Y. Result Turnaround Time of RT-PCR for SARS-CoV-2 is the Main Cause of COVID-19 Diagnostic Delay: A Country-Wide Observational Study of Mexico and Colombia. Rev Invest Clin. 2022;74: 071–080. doi:10.24875/RIC.21000542

22. Díaz LA, García-Salum T, Fuentes-López E, Reyes D, Ortiz J, Chahuan J, et al. High prevalence of SARS-CoV-2 detection and prolonged viral shedding in stools: A systematic review and cohort study. Gastroenterol Hepatol. 2022;45: 593–604. doi:10.1016/j.gastrohep.2021.12.009

23. Cerrada-Romero C, Berastegui-Cabrera J, Camacho-Martínez P, Goikoetxea-Aguirre J, Pérez-Palacios P, Santibáñez S, et al. Excretion and viability of SARS-CoV-2 in feces and its association with the clinical outcome of COVID-19. Sci Rep. 2022;12. doi:10.1038/s41598-022-11439-7

24. Lavania M, Joshi MS, Ranshing SS, Potdar VA, Shinde M, Chavan N, et al. Prolonged Shedding of SARS-CoV-2 in Feces of COVID-19 Positive Patients: Trends in Genomic Variation in First and Second Wave. Front Med (Lausanne). 2022;9. doi:10.3389/fmed.2022.835168

25. Cavany S, Bivins A, Wu Z, North D, Bibby K, Perkins TA. Inferring SARS-CoV-2 RNA shedding into wastewater relative to the time of infection. Epidemiol Infect. 2022;150. doi:10.1017/S0950268821002752

26. Prasek SM, Pepper IL, Innes GK, Slinski S, Betancourt WQ, Foster AR, et al. Variant-specific SARS-CoV-2 shedding rates in wastewater. Science of the Total Environment. 2023;857. doi:10.1016/j.scitotenv.2022.159165

27. Iuliano AD, Roguski KM, Chang HH, Muscatello DJ, Palekar R, Tempia S, et al. Estimates of global seasonal influenza-associated respiratory mortality: a modelling study. The Lancet. 2018;391: 1285–1300. doi:10.1016/S0140-6736(17)33293-2

28. Fleming DM, Taylor RJ, Haguinet F, Schuck-Paim C, Logie J, Webb DJ, et al. Influenza-attributable burden in United Kingdom primary care. Epidemiol Infect. 2016;144: 537–547. doi:10.1017/S0950268815001119

29. Romanelli RJ, Cabling ML, Marciniak-Nuqui Z, Marjanovic S, Morris S, Dufresne E, et al. The societal and indirect economic burden of seasonal influenza in the United Kingdom. 2023. Available: www.rand.org/about/principles.

30. Salisbury D, Ramsay M, Noakes K. Chapter 19 Influenza. Green Book - Immunisation against infectious disease. Department of Health; 2023.

31. Biggerstaff M, Cauchemez S, Reed C, Gambhir M, Finelli L. Estimates of the reproduction number for seasonal, pandemic, and zoonotic influenza: A systematic review of the literature. BMC Infect Dis. 2014;14. doi:10.1186/1471-2334-14-480

32. Puhach O, Meyer B, Eckerle I. SARS-CoV-2 viral load and shedding kinetics. Nature Reviews Microbiology. Nature Research; 2023. pp. 147–161. doi:10.1038/s41579-022-00822-w

33. Wolfe MK, Duong D, Bakker KM, Ammerman M, Mortenson L, Hughes B, et al. Wastewater-Based Detection of Two Influenza Outbreaks. Environ Sci Technol Lett. 2022;9: 687–692. doi:10.1021/acs.estlett.2c00350

34. Li Y, Wang X, Blau DM, Caballero MT, Feikin DR, Gill CJ, et al. Global, regional, and national disease burden estimates of acute lower respiratory infections due to respiratory syncytial virus in children younger than 5 years in 2019: a systematic analysis. The Lancet. 2022;399: 2047–2064. doi:10.1016/S0140-6736(22)00478-0

35. Fleming DM, Taylor RJ, Lustig RL, Schuck-Paim C, Haguinet F, Webb DJ, et al. Modelling estimates of the burden of Respiratory Syncytial virus infection in adults and the elderly in the United Kingdom. BMC Infect Dis. 2015;15. doi:10.1186/s12879-015-1218-z

36. Wilkinson T, Beaver S, Macartney M, McArthur E, Yadav V, Lied-Lied A. Burden of respiratory syncytial virus in adults in the United Kingdom: A systematic literature review and gap analysis. Influenza and other Respiratory Viruses. John Wiley and Sons Inc; 2023. doi:10.1111/irv.13188

37. Reis J, Shaman J. Retrospective Parameter Estimation and Forecast of Respiratory Syncytial Virus in the United States. PLoS Comput Biol. 2016;12. doi:10.1371/journal.pcbi.1005133

38. Pires SM, Fischer-Walker CL, Lanata CF, Devleesschauwer B, Hall AJ, Kirk MD, et al. Aetiology-specific estimates of the global and regional incidence and mortality of diarrhoeal diseases commonly transmitted through food. PLoS One. 2015;10. doi:10.1371/journal.pone.0142927

39. Gaythorpe KAM, Trotter CL, Lopman B, Steele M, Conlan AJK. Norovirus transmission dynamics: A modelling review. Epidemiology and Infection. Cambridge University Press; 2018. pp. 147–158. doi:10.1017/S0950268817002692

40. Atmar RL, Opekun AR, Gilger MA, Estes MK, Crawford SE, Neill FH, et al. Norwalk virus shedding after experimental human infection. Emerg Infect Dis. 2008;14: 1553–1557. doi:10.3201/eid1410.080117

41. Pecson BM, Darby E, Haas CN, Amha YM, Bartolo M, Danielson R, et al. Reproducibility and sensitivity of 36 methods to quantify the SARS-CoV-2 genetic signal in raw wastewater: Findings from an interlaboratory methods evaluation in the U.S. Environ Sci (Camb). 2021;7: 504–520. doi:10.1039/d0ew00946f

42. Palmer EJ, Maestre JP, Jarma D, Lu A, Willmann E, Kinney KA, et al. Development of a reproducible method for monitoring SARS-CoV-2 in wastewater. Science of the Total Environment. 2021;799. doi:10.1016/j.scitotenv.2021.149405

43. Klymus KE, Merkes CM, Allison MJ, Goldberg CS, Helbing CC, Hunter ME, et al. Reporting the limits of detection and quantification for environmental DNA assays. Environmental DNA. 2020;2: 271–282. doi:10.1002/edn3.29

44. Parra-Guardado AL, Sweeney CL, Hayes EK, Trueman BF, Huang Y, Jamieson RC, et al. Development of a rapid pre-concentration protocol and a magnetic beads-based RNA extraction method for SARS-CoV-2 detection in raw municipal wastewater. Environ Sci (Camb). 2022;8: 47–61. doi:10.1039/d1ew00539a

45. Ahmed W, Bertsch PM, Angel N, Bibby K, Bivins A, Dierens L, et al. Detection of SARS-CoV-2 RNA in commercial passenger aircraft and cruise ship wastewater: A surveillance tool for assessing the presence of COVID-19 infected travellers. J Travel Med. 2021;27: 1–11. doi:10.1093/JTM/TAAA116

46. Sokolova E. Norovirus Dynamics in Wastewater Discharges and in the Recipient Drinking Water Source : Long-Term Monitoring and Hydrodynamic. 2016;50: 10851–10858.

47. Huang Y, Zhou N, Zhang S, Yi Y, Han Y, Liu M, et al. Norovirus detection in wastewater and its correlation with human gastroenteritis : a systematic review and meta - analysis. Environmental Science and Pollution Research. 2022; 22829–22842. doi:10.1007/s11356-021-18202-x

48. Toribio-Avedillo D, Gómez-Gómez C, Sala-Comorera L, Rodríguez-Rubio L, Carcereny A, García-Pedemonte D, et al. Monitoring influenza and respiratory syncytial virus in wastewater. Beyond COVID-19. Science of the Total Environment. 2023;892. doi:10.1016/j.scitotenv.2023.164495

49. Stadler LB, Ensor KB, Clark JR, Kalvapalle P, LaTurner ZW, Mojica L, et al. Wastewater Analysis of SARS-CoV-2 as a Predictive Metric of Positivity Rate for a Major Metropolis. medRxiv. 2020; 1–36. doi:https://doi.org/10.1101/2020.11.04.20226191

50. Hassard F, Vu M, Rahimzadeh S, Castro-Gutierrez V, Stanton I, Burczynska B, et al. Wastewater monitoring for detection of public health markers during the COVID-19 pandemic: Near-source monitoring of schools in England over an academic year. PLoS One. 2023;18. doi:10.1371/journal.pone.0286259

51. Hakki S, Zhou J, Jonnerby J, Singanayagam A, Barnett JL, Madon KJ, et al. Onset and window of SARS-CoV-2 infectiousness and temporal correlation with symptom onset: a prospective, longitudinal, community cohort study. Lancet Respir Med. 2022;10: 1061–1073. doi:10.1016/S2213-2600(22)00226-0

52. Mellou K, Sapounas S, Panagoulias I, Gkova M, Papadima K, Andreopoulou A, et al. Time Lag between COVID-19 Diagnosis and Symptoms Onset for Different Population Groups: Evidence That Self-Testing in Schools Was Associated with Timely Diagnosis among Children. Life. 2022;12. doi:10.3390/life12091305

53. COVID-19: general public testing behaviours - GOV.UK. [cited 1 Feb 2024]. Available: https://www.gov.uk/government/publications/lfd-tests-how-and-why-they-were-used-during-the-pandemic/covid-19-general-public-testing-behaviours

54. Whitaker M, Elliott J, Bodinier B, Barclay W, Ward H, Cooke G, et al. Variant-specific symptoms of COVID-19 in a study of 1,542,510 adults in England. Nat Commun. 2022;13. doi:10.1038/s41467-022-34244-2

55. Singer AC, Thompson JR, Filho CRM, Street R, Li X, Castiglioni S, et al. A world of wastewater-based epidemiology. Nature Water. 2023;1: 408–415. doi:10.1038/s44221-023-00083-8
